# Global, regional, and national burden of smoking-attributable digestive cancers in adults aged ≥60 years, 1990–2021, with projections to 2036: A secondary dataset analysis of Global Burden of Disease (GBD) 2021

**DOI:** 10.18332/tid/216110

**Published:** 2026-02-17

**Authors:** Xiaoyi Wang, Liuye Huang

**Affiliations:** 1 Qingdao Medical College of Qingdao University, Qingdao, People’s Republic of China; 2 Department of Gastroenterology, Yantai Yuhuangding Hospital, Yantai, People’s Republic of China

**Keywords:** digestive system cancers, older adults, Global Burden of Disease, decomposition analysis, Socio-demographic Index

## Abstract

**INTRODUCTION:**

Smoking is a key driver of esophageal, stomach, liver, pancreatic, and colorectal cancers, and its impact is stronger in adults aged ≥60 years due to cumulative exposure and population aging. A comprehensive analysis of temporal trends and sociodemographic drivers for major digestive cancers in this population is lacking. This study quantified smoking-attributable deaths and disability-adjusted life years (DALYs) from digestive cancers in adults aged ≥60 years globally from 1990 to 2021. It also projected future burdens to 2036 to inform age-focused tobacco control and cancer prevention.

**METHODS:**

This study is a secondary dataset analysis of data from GBD 2021. We analyzed smoking-attributable deaths and DALYs for esophageal, stomach, liver, pancreatic, and colorectal cancers among adults aged ≥60 years across 204 countries and territories (1990–2021). We decomposed temporal changes into contributions from population growth, aging, and changes in age-specific rates. Associations with the sociodemographic index (SDI) were assessed, and future burdens (2022–2036) were projected using a Bayesian age-period-cohort model.

**RESULTS:**

From 1990 to 2021, global smoking-attributable deaths increased by 27.5% (398426 to 508147) and DALYs by 14.6% (8.9 to 10.2 million). Decomposition attributed the increase primarily to population growth (41–43%), partially offset by declining age-specific rates (-30% to -35%). Regional disparities were stark: high-SDI regions stabilized burdens through epidemiological improvements, while low-SDI regions saw population-driven escalations. SDI was positively correlated with burden at the country level (ρ=0.20–0.48; p<0.001), peaking at mid-high SDI.

**CONCLUSIONS:**

Despite falling age-specific rates, the absolute burden of smoking-attributable digestive cancers in older adults is rising in parallel with demographic growth. Projections to 2036 suggest that, if current demographic trends continue, absolute deaths and DALYs are expected to continue increasing as populations age and grow.

## INTRODUCTION

Tobacco smoking remains the leading preventable cause of cancer worldwide, responsible for approximately 22% of global cancer deaths and contributing to malignancies across multiple anatomical sites^1^. Digestive system cancers, encompassing esophageal (EC), stomach (SC), liver (LC), pancreatic (PC), and colorectal (CRC) malignancies, represent a substantial proportion of smoking-attributable morbidity and mortality, a causal relationship firmly established through decades of epidemiological and mechanistic research^2^. The carcinogenic pathways involve direct exposure to tobacco-derived compounds through the intake of smoke and saliva, systemic circulation of metabolites, and complex interactions with organ-specific risk factors, including infectious agents, dietary patterns, and genetic susceptibilities^3^. The global demographic transition toward population aging has profound implications for cancer epidemiology, particularly for smoking-related malignancies, where cumulative exposure effects are amplified by age-related immune senescence, reduced DNA repair capacity, and increased cellular oxidative stress^4^. Populations aged ≥60 years represent the fastest-growing demographic segment globally, projected to reach 2.1 billion by 2036, with smoking-attributable cancer risks escalating exponentially in this age group due to latency periods spanning decades between initial exposure and clinical manifestation^5^. Furthermore, elderly populations often exhibit complex comorbidity profiles and medication interactions that complicate both prevention and treatment strategies, necessitating specialized approaches to burden reduction^6^.

Current global health initiatives, including the WHO Framework Convention on Tobacco Control and the Sustainable Development Goals, emphasize reducing premature mortality from non-communicable diseases, yet implementation progress varies dramatically across socioeconomic contexts^7^. High-income countries have achieved substantial reductions in smoking prevalence and associated cancer rates through comprehensive tobacco control policies, early detection programs, and advanced treatment modalities. However, low- and middle-income countries face persistent challenges, including limited healthcare infrastructure, competing infectious disease priorities, and tobacco industry marketing strategies targeting vulnerable populations^8^. These disparities are further compounded by varying stages of the epidemiological transition, where some regions experience rising cancer burdens due to lifestyle changes and increased longevity, while others benefit from prevention and treatment advance^9^. Despite extensive research on individual digestive cancers and their relationship to smoking, comprehensive analyses examining all major smoking-attributable digestive malignancies collectively in elderly populations, remain scarce.

A previous study focused on specific cancer types, broader adult age ranges, or regional contexts, which limits its ability to inform global patterns and their underlying drivers^10^. Moreover, while aggregate burden estimates are routinely reported, decomposition analyses that separate the contributions of population growth, demographic aging, and epidemiological changes provide crucial insights for policy prioritization and resource allocation, yet have been underutilized in tobacco-related cancer research^11,12^.

Understanding the complex relationships between socioeconomic development and cancer burdens is essential for designing effective interventions tailored to different developmental contexts. The sociodemographic index (SDI), a composite measure incorporating income per capita, education level, and fertility rates, provides a validated framework for examining these associations and identifying development thresholds where burden trajectories may shift^13^. However, systematic analyses of SDI correlations with smoking-attributable digestive cancer burdens in elderly populations across global contexts remain limited, representing a significant knowledge gap for evidence-based policy development.

This study addresses these limitations by providing, a comprehensive decomposition and correlation analysis of smoking-attributable burdens for major digestive system cancers among populations aged ≥60 years, using the Global Burden of Disease (GBD) 2021 dataset spanning 204 countries and territories from 1990 to 2021^1,11,14^. This study quantifies demographic and epidemiological contributions to inform targeted strategies for reducing smoking-attributable digestive cancer burden in aging populations across socioeconomic development levels.

## METHODS

### Data acquisition and sources

This is a secondary analysis of the Global Burden of Disease Study 2021 (GBD 2021) data. We used the GBD 2021 dataset to assess smoking-attributable digestive system cancers among populations aged ≥60 years. Data were obtained via the Global Health Data Exchange (GHDx) Results Tool, which provides open-access global and regional health metrics. GBD 2021 covers 371 diseases and injuries with estimates of incidence, prevalence, mortality, and disability-adjusted life years (DALYs), as well as 88 risk factors across behavioral, environmental, occupational, and metabolic domains^1^. Our analysis focused on smoking-attributable mortality and DALYs for five major digestive system cancers: EC, SC, LC, PC, and CRC in adults aged ≥60 years worldwide. In this study, smoking-attributable estimates were based on the GBD level 3 risk factor ‘smoking’, which captures active tobacco smoking (current and former smokers of any smoked tobacco products). Secondhand smoke exposure is modeled as a separate risk factor in GBD and was not included in our definition of smoking exposure. Population attributable fractions (PAFs) and smoking-attributable deaths and DALYs for adults aged ≥60 years were taken directly from the GBD 2021 comparative risk assessment results for the level 3 risk factor ‘smoking’, rather than being recalculated from primary exposure and relative risk data. We relied on the standard GBD counterfactual risk assessment framework as described previously^1^. DALYs, defined as the sum of years lived with disability (YLDs) and years of life lost (YLLs) due to premature mortality, were used to capture total disease burden^1,11^. The SDI is a composite measure ranging from 0 to 1, derived from a region’s total fertility rate for those aged ≤25 years, mean years of education among individuals aged ≥15 years, and lag-distributed per capita income. Based on this metric, locations are categorized into five SDI quintiles: low, low-middle, middle, high-middle, and high^13,14^. We analyzed data for 204 countries and territories from 1990 to 2021 to provide temporal trends and a comprehensive assessment of the global burden of smoking-attributable digestive system cancers in older adults. We did not identify any material missingness in the extracted GBD 2021 estimates for the included strata, so no missing-data imputation was performed. Ethical approval and informed consent were not required because only publicly available, de-identified data were used. Reporting followed the GATHER guidelines^15^.

### Population analysis and global burden analysis

We quantified age-standardized mortality rates (ASMRs), age-standardized DALY rates (ASDRs), and their 95% uncertainty intervals (UIs) for smoking-attributable digestive system cancers among adults aged ≥60 years using GBD 2021 estimates across 204 countries and territories, 22 GBD regions, and five SDI quintiles^1^. Outcomes were stratified by gender (male and female) and by eight elderly age subgroups to capture granular age-specific patterns within older populations. This stratification encompassed the following age brackets: 60–64, 65–69, 70–74, 75–79, 80–84, 85–89, 90–94, and ≥95 years. To characterize geographical heterogeneity, we produced high-resolution choropleth maps depicting the global burden of smoking-attributable digestive cancers and disparities across sociodemographic and regional contexts, highlighting priority areas for targeted interventions. Temporal trends in ASMRs and ASDRs from 1990 to 2021 were assessed using estimated annual percentage changes (EAPCs), derived from log-linear regression of ln (age-standardized rate) on calendar year (Supplementary file Equation S1). EAPCs were calculated using log-linear regression models and are presented with 95% confidence intervals (CIs), while GBD-derived estimates are reported with 95% uncertainty intervals (UIs) and Bayesian projections with 95% credible intervals (CrIs). Joinpoint regression was not applied; temporal trends were evaluated solely using EAPCs derived from log-linear regression models. To delineate distinct epidemiological patterns and burden trajectories, we performed cancer-specific analyses focusing on five digestive cancers: esophageal, stomach, liver, pancreatic, and colorectal.

### Decomposition analysis

We conducted a decomposition analysis using the Das Gupta stepwise replacement method to quantify the contributions of population growth, population aging, and epidemiological change to changes in the absolute numbers of smoking-attributable deaths and DALYs from 1990 to 2021. Decomposition was performed on counts rather than age-standardized rates; trends in age-standardized rates were assessed separately using EAPCs. Following established GBD analytic conventions, the total change in burden was partitioned into three components: 1) population growth, reflecting increases due to larger absolute population size; 2) population aging, reflecting shifts in age structure toward older demographics; and 3) epidemiological change, reflecting alterations in age-specific rates independent of demographic shifts^11,16^. The decomposition of changes in the number of smoking-attributable deaths and DALYs followed the identity shown in Supplementary file Equation S2.

We implemented the decomposition separately by cancer type, sex, and SDI quintile to delineate subgroup-specific patterns. This approach clarifies whether increases in burden are predominantly attributable to demographic expansion or to adverse shifts in age-specific risk, thereby informing priorities for prevention and control^16^.

### SDI correlation analysis

We assessed the association between socioeconomic development and smoking-attributable digestive cancer burdens through SDI-based correlation analyses at both country and regional levels. At the country level, we computed Spearman rank correlation coefficients (ρ) between 2021 SDI values and age-standardized mortality and DALY rates for each cancer type across all 204 countries and territories. Because SDI and burden metrics showed skewed distributions and potentially monotonic but non-linear relationships, we used Spearman’s rank correlation rather than Pearson’s product–moment correlation to assess associations between SDI and ASMRs/ASDRs and between SDI and estimated annual percentage changes (EAPCs). Statistical significance was evaluated using two-tailed tests with α=0.05, and the Bonferroni correction was applied for multiple comparisons across the five cancer types. For regional analyses, we aggregated country-level estimates within each of the 22 GBD regions using population-weighted averages to ensure representativeness. We visualized SDI–burden relationships with scatter plots and fitted lines to screen for nonlinearity. To flexibly capture complex or non-monotonic patterns potentially indicative of differing stages of epidemiological transition, we applied locally weighted scatterplot smoothing (LOWESS) to the regional data^13,14^.

### Prediction analysis

To inform policy and resource planning, we stratified analyses by sex (female, male) and applied a Bayesian age-period-cohort (BAPC) modeling framework from 2022 to 2036. The model integrates age-specific patterns and temporal (period) variation to project future burdens with uncertainty, offering a comprehensive outlook for planning. We implemented BAPC using integrated nested Laplace approximation (INLA) to obtain accurate approximations to marginal posterior distributions while avoiding the mixing and convergence challenges common with Markov chain Monte Carlo (MCMC) methods. Priors for smoothness were specified via second-order random-walk (RW2) structures on age, period, and cohort effects, with hyperparameters estimated from the data. Model performance was evaluated through out-of-sample cross-validation and posterior predictive checks. Forecasts are presented as posterior medians with 95% credible intervals^17,18^.

In greater detail, for each cancer type and sex, we modeled age-specific smoking-attributable deaths and DALYs using 5-year GBD age groups among adults aged ≥60 years (60–64, 65–69, 70–74, 75–79, 80–84, 85–89, 90–94, and ≥95 years). Calendar years from 1990 to 2021 were treated as periods and used as training data, and projections were generated for 2022–2036, consistent with the primary analyses. We modeled age- and period-specific smoking-attributable counts using a Poisson log-linear age-period-cohort model, with log (population) as an offset. We report posterior predictive medians and 95% credible intervals, and projected ASMRs/ASDRs were obtained via direct standardization to the GBD standard population. Model fit and predictive performance were assessed using information criteria and a hold-out validation, comparing observed versus fitted values in the most recent years.

### Statistical software

The study utilized a suite of statistical and geospatial tools for data analysis. For statistical modeling, we employed R (version 4.3.3; R Foundation for Statistical Computing, Vienna, Austria)^19^ and Stata (version 18; StataCorp LLC, College Station, TX, USA)^20^, developing custom scripts for decomposition, correlation, and sensitivity analyses. Spatial patterns were analyzed and visualized using ArcGIS Pro (version 3.0; Esri, Redlands, CA, USA)^21^ and QGIS (version 3.28; QGIS Development Team)^22^, which enabled the creation of high-resolution choropleth maps to display global burden and disparities. Finally, data visualization was performed in R to generate forest plots, scatter plots, and multi-panel figures. Time series analyses and EAPC calculations were also conducted in R^23^.

### Statistical significance

Statistical significance was assessed using two-sided p-values with p<0.05 considered significant. For sets of related tests (e.g. correlations across the five cancer types), we accounted for multiple comparisons but did not apply a single, strict Bonferroni-adjusted threshold across all subgroup- and region-specific analyses. P-values are therefore treated as descriptive and interpreted in relation to effect sizes and the overlap or non-overlap of 95% uncertainty intervals. Given the large number of subgroups and location-specific comparisons, we did not perform exhaustive pairwise hypothesis testing across all strata (sex, SDI quintile, age group, and region). Instead, we focused on the magnitude and direction of differences in ASMRs, ASDRs, and EAPCs, evaluated against their 95% uncertainty intervals. Credibility intervals (CrIs) for model-based estimates and UIs for GBD-derived quantities were calculated at the 95% level, with UIs from GBD data reflecting both parameter and model uncertainty in the underlying estimation process^15,24^.

## RESULTS

### Global burden of smoking-attributable digestive system cancers among adults aged _≥_60 years (1990–2021)

In 2021, total smoking-attributable DALYs among older adults were: EC, 3268824 (95% UI: 2663716–3802166); liver cancer, 708190 (95% UI: 240659–1196883); PC, 1102207 (95% UI: 943669–1273484); gastric cancer, 1674301 (95% UI: 1282517–2170914); and CRC, 727595 (95% UI: 451515–1021401). The corresponding ASDRs per 100000 were 298.69 (95% UI: 240.45–360.12) for esophageal, 64.33 (95% UI: 21.83–108.81) for liver, 100.44 (95% UI: 85.88–116.20) for pancreatic, 153.26 (95% UI: 117.34–198.66) for gastric, and 66.51 (95% UI: 41.24–93.47) for CRC. Compared with 1990, DALYs rose by 57.5% (esophageal), 83.1% (liver), 94.0% (pancreatic), 2.8% (gastric), and 58.0% (colorectal). EAPCs in ASDRs confirmed downward trends: -1.18 (95% CI: -1.25 – -1.10) for esophageal, -0.69 (95% CI: -0.82 – -0.56) for liver, -0.37 (95% CI: -0.42 – -0.32) for pancreatic, -2.48 (95% CI: -2.53 – -2.44) for gastric, and -1.19 (95% CI: -1.23 – -1.16) for CRC (Supplementary file Tables S1–S10).

### Global burden of disease and trends in digestive system cancers attributable to smoking by sex and age

Regarding sex, deaths, DALYs, and their age-standardized rates (ASRs) for smoking-attributable EC, LC, PC, SC, and CRCs have remained consistently higher in men than in women over the long-term, reflecting historically greater smoking prevalence and exposure among males. In 2021, among adults aged ≥60 years, male-to-female mortality ratios were consistently elevated across major gastrointestinal cancers. These ratios showed a pronounced male predominance: approximately 6.3:1 for EC (24892 vs 3940 deaths), 2.6:1 for LC (22202 vs 8588), 3.4:1 for PC (38020 vs 11174), 4.9:1 for SC (68540 vs 14116), and 2.9:1 for CRC (23546 vs 8070). Corresponding DALY ratios were similarly elevated at 6.4:1 for EC (577145 vs 90274), 2.7:1 for LC (735625 vs 276956), 3.4:1 for PC (774545 vs 226654), 4.9:1 for SC (1344196 vs 274739), and 2.9:1 for CRC (491730 vs 167801). Although these disparities have persisted from 1990 to 2021, the gender gap in ASRs has narrowed slightly, particularly in high-income regions where male smoking has declined. (Supplementary file Figure S1).

From 1990 to 2021, absolute deaths and DALYs from all five cancers rose among adults aged ≥60 years, paralleling global population aging and patterns of cumulative long-term smoking exposure described in prior studies. The steepest rises occurred in those aged 75–89 years; for instance, SC deaths in this band increased from about 25000 in 1990 to over 45000 in 2021, accounting for nearly half of the total increase across ages. Similar patterns were observed across cancers, with PC and CRCs showing the highest proportional growth in ages 80–84 and 85–89 years (about 120–150% increases), likely compounded by comorbidities and delayed diagnosis. Despite rising counts, ASMRs and ASDRs generally declined relative to 1990 across age bands, with the most pronounced improvements in the younger elderly (60–69 years), where EAPCs ranged roughly from -2.5 to -1.0 for most cancers, a pattern consistent with concurrent advances in prevention and treatment access. However, small late-life reversals were observed: in CRC, ASMRs rose slightly from 4.5 to 4.8 per 100000 and ASDRs from 95 to 102 per 100000 among those aged >80 years in 2021 versus 1990, potentially linked to rising obesity and dietary risks interacting with lifelong smoking; LC ASDRs also edged up among those aged ≥90 years (EAPC +0.2) (Supplementary file Figure S2).

### Global burden of disease and trends in digestive system cancers attributable to smoking in different SDI levels

From 1990 to 2021, pronounced disparities were evident in the smoking-attributable burden of digestive system cancers across sociodemographic index (SDI) strata. High SDI regions consistently showed the lowest ASMRs and ASDRs for all major digestive cancers, whereas low and low-middle SDI groups bore the highest burdens throughout. In 2021, for example, the male ASMR for smoking-attributable EC was about 3.7 per 100000 in high SDI regions versus over 8.0 per 100000 in low SDI regions. The patterns observed for DALYs closely mirrored those for mortality. High-SDI regions, which began with lower ASDRs in 1990, subsequently experienced the most substantial declines. This was evidenced by a reduction of >50% in SC DALY rates in these regions. In contrast, many low and low-middle SDI settings demonstrated only modest improvements or a trend toward a plateau. Despite improvements in age-standardized metrics, absolute deaths and DALYs increased in most SDI groups, especially in low and middle-SDI regions, reflecting population growth and aging; for instance, low-middle SDI regions experienced substantial rises in liver and PC deaths, whereas high SDI regions maintained stable or slightly declining counts. Across all SDI categories and time points, males experienced substantially higher burdens than females, with male-to-female ratios for both death and DALY rates typically exceeding 2:1 and remaining relatively stable over three decades (Supplementary file Figure S3).

### Global burden of disease and trends in digestive system cancers attributable to smoking in different regions

From 1990 to 2021, East Asia and South Asia consistently contributed the largest absolute numbers of smoking-attributable deaths and DALYs from digestive system cancers, driven by large and aging populations alongside persistently high smoking prevalence. In contrast, high-income regions such as high-income North America and Western Europe achieved the steepest declines in ASMR and DALY rates (ASDRs) owing to robust tobacco control, earlier detection, and better treatment access. In 1990, historically high ASMRs and ASDRs for esophageal and liver cancers were observed in regions with entrenched tobacco epidemics, notably East Asia and Central Europe. Driven by decades of prior smoking prevalence, the rates in these areas frequently surpassed 15–25 per 100000. Over the subsequent three decades, comprehensive public health measures in affluent areas substantially reversed these trends, while many low- and middle-income regions, including parts of Sub-Saharan Africa and the Caribbean, experienced slower declines or plateaus by 2021; for example, PC rates in Eastern Sub-Saharan Africa hovered around 8–12 per 100000 versus below 4 per 100000 in Australasia. Despite widespread improvements in age-standardized metrics, the absolute numbers of deaths and DALYs rose in most regions (Supplementary file Figure S4).

Regional heterogeneity remains pronounced. East Asia continues to bear the heaviest absolute burden due to its sizable elderly population and smoking behaviors. By 2021, Western Europe registered consistently among the lowest ASMRs and ASDRs across digestive cancers, a trend attributable to comprehensive smoke-free policies and mature screening programs. These interventions facilitated sharp declines, as seen in CRC, where the ASDRs fell from roughly 150 to about 70 per 100000, corresponding to EAPCs between -2.5 and -1.8. While in South Asia, absolute DALYs saw notable surges; for instance, LC rose from approximately 120000 in 1990 to 210000 in 2021, highlighting the persistent middle-income challenges in the context of rapid industrialization. From 1990 to 2021, a rising burden of cancer deaths and DALYs was observed across many low- and middle resource settings. Southeast Asia and Central Asia maintained the highest SC ASMRs through 2021, at approximately 10–15 per 100000. Meanwhile, Sub-Saharan Africa, specifically Eastern and Southern subregions, saw the slowest progress against EC, with ASDRs declining only marginally from about 180 to 160 per 100000 (EAPC near -0.5), a challenge compounded by resource constraints and overlapping HIV epidemics (Supplementary file Tables S1 and S2). This period also saw some of the sharpest increases in the Asia-Pacific, exemplified by a more than 90% rise in PC deaths in South-East Asia, from roughly 10000 to 19000. While a broad global decline in ASRs was evident, its magnitude varied: high-income North America achieved substantial reductions (e.g. LC ASMR from about 5.5 to 3.2 per 100000), whereas Andean Latin America and Oceania showed minor rises in certain ASDRs (e.g. EAPC +0.2 for SC), reflecting vulnerabilities in geographically isolated or economically transitional contexts (Supplementary file Figure S5).

### Global burden of disease and trends in digestive system cancers attributable to smoking in 204 countries and territories

From 1990 to 2021, the largest declines in ASMRs and ASDRs for smoking-attributable digestive cancers were achieved by countries that implemented comprehensive tobacco control and robust screening programs. Leading examples include Australia, Canada, the United States, Germany, and the United Kingdom, where reductions often exceeded 50–70% across most cancer sites. In contrast, progress was slower or reversed in parts of Sub-Saharan Africa, the Caribbean, and Central Asia, where weak enforcement, socioeconomic constraints, and rising initiation yielded EAPCs near zero or positive. Declines also varied by cancer within countries: Japan saw steep drops in SC (from ~15.2 to ~5.1 per 100000; EAPC -3.5) but more modest decreases for EC, influenced by alcohol and cultural factors. China experienced rapid declines in LC and CRC rates. This progress was second only to leading nations such as Australia and Sweden for liver cancer (EAPC -2.8), and Brazil for CRC, where rates fell from approximately 9.5 to 4.5 per 100000. In contrast, PC incidence rose from about 3.5 to 5.2 per 100000 (EAPC +0.9). Mongolia and Thailand exhibit some of the highest esophageal and SC rates (e.g. esophageal in Mongolia >20 per 100000 in 2021). India, Russia, and South Africa remain among the highest for liver and PCs (e.g. liver in India ~11–14 per 100000). For CRC, Hungary, Cuba, and Bolivia rank highest. Marked gender disparities in cancer burden persist. For instance, men in Russia and India face ASMRs for EC and LC that are three to five times higher than those of women. Geographically, deaths and DALYs are heavily concentrated in a few populous nations, namely China, India, the United States, and Indonesia. These four countries collectively account for a dominant share of global digestive cancer deaths, including 52.1% of liver, 60.4% of esophageal, 68.9% of stomach, 55.7% of pancreatic, and 62.3% of colorectal cancer deaths, with closely corresponding shares for DALYs (Supplementary file Tables S1–S10).

### Decomposition analysis of smoking-attributable burden of digestive system cancers among populations aged _≥_60 years

**Figure 1:**
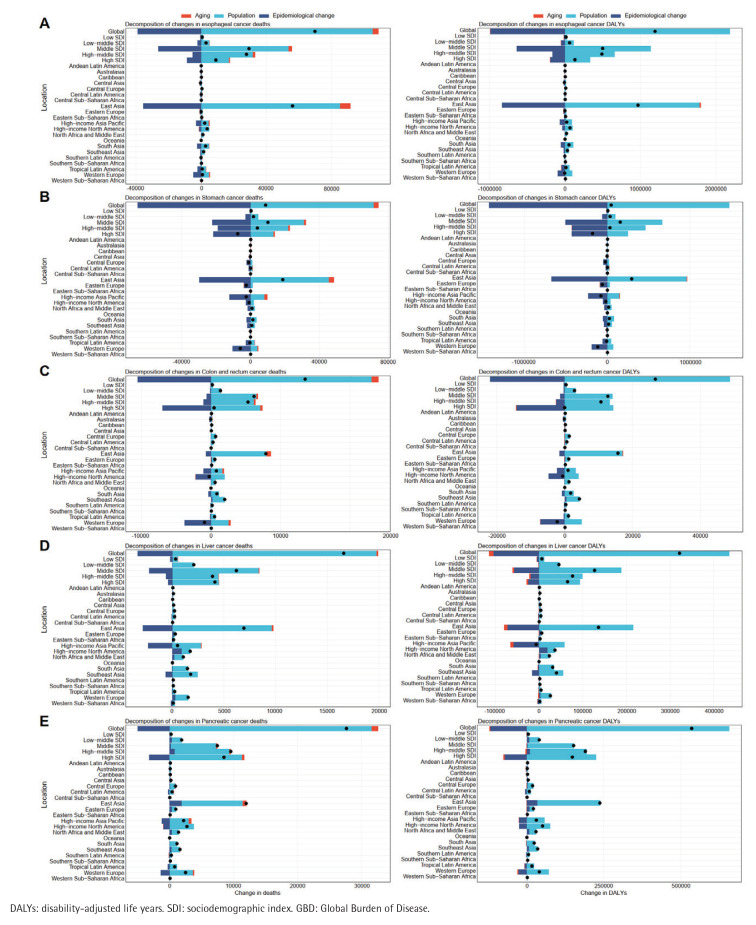
Decomposition of changes in smoking-attributable deaths and DALYs from five digestive cancers in adults aged ≥60 years, 1990–2021, by SDI quintile and GBD region. Panels show (A) esophageal cancer, (B) stomach cancer, (C) colon and rectum cancer, (D) liver cancer, and (E) pancreatic cancer. For each cancer, the left panel shows the contributions of population growth, population aging, and epidemiological change to deaths, and the right panel shows the corresponding contributions to DALYs

Decomposition analyses revealed marked heterogeneity in the drivers of smoking-attributable digestive system cancer burden among adults aged ≥60 years. From 1990 to 2021, population growth was the dominant contributor, accounting for approximately 41.27% of deaths and 43.15% of DALYs across EC, SC, LC, PC, and CRCs, followed by epidemiological transitions (29.86% of deaths; 33.47% of DALYs). Aging had a comparatively smaller overall impact, typically 5–15% across strata. These patterns, derived from aggregated SDI-level data, underscore how demographic shifts in older cohorts amplify the effects of cumulative smoking exposure and age-related vulnerabilities. Drivers varied by SDI level. In high-SDI regions (e.g. high-income North America, Western Europe), population dynamics remained the leading driver of deaths (about 35–50%), alongside reduced epidemiological influence (15–25%) and a stronger aging effect (up to 20%), yielding stable or declining net burdens due to mature tobacco control and geriatric care. As SDI decreased, the contribution of epidemiological transitions grew (reaching 40–50% in low-SDI settings such as Sub-Saharan Africa), while population factors continued to dominate (50–70%), aligning with rising age-specific death rates amid limited access to cessation services and screening. For DALYs, the population contribution declined with higher SDI (from roughly 60% in low-SDI to about 30% in high-SDI), whereas aging became more influential (15–25% in high-SDI), reflecting longer survival accompanied by greater morbidity in older populations. Epidemiological transitions peaked in middle- and middle-high-SDI regions (35–45%): transitional economies, such as those in East Asia, achieved rapid rate reductions for liver and ECs, but these gains were partially offset by rapid aging and population expansion (Figure 1).

### SDI correlation analysis

Supplementary file Figure S6 illustrates the relationship between SDI and smoking-attributable deaths and DALYs from digestive system cancers at national and regional scales. At the country level, a broadly positive correlation was observed: age-standardized death and DALY rates tended to rise with increasing SDI, peaking when SDI ranged from 0.70 to 0.85, particularly for colorectal and pancreatic cancer. This indicates that more developed countries often bear higher absolute burdens of smoking-attributable digestive cancers. The correlation coefficients (ρ) between SDI and deaths were 0.28 (EC), 0.27 (SC), 0.15 (LC; p=0.032), 0.79 (PC), and 0.65 (CRC), all other p<0.001, indicating moderate associations. Corresponding ρ values for DALYs were 0.25, 0.23, 0.15 (p=0.029), 0.78, and 0.66 (all other p<0.001). Across the SDI spectrum, the smoothed curves showed an overall ‘M-shaped’ relationship: deaths and DALYs rose sharply with SDI when SDI was below 0.45 and again between 0.65 and 0.80, but declined when SDI exceeded 0.80 and in the intermediate range of 0.45–0.65. This complexity likely reflects heterogeneity in age structures, healthcare access, effectiveness of tobacco control, and lagged effects of historical smoking.

### Predicted trends in 2036

Using an age–period–cohort projection framework analogous to BAPC, our forecasts indicate that by 2036, the global absolute deaths and DALYs from smoking-attributable digestive cancers in adults aged ≥60 years will continue to increase, driven by population growth and aging, whereas ASMRs are expected to decline across major sites (esophagus, stomach, liver, pancreas, and colorectum) over the subsequent 15 years (to 2036) reflecting ongoing risk reduction and treatment improvements. Specifically, site-wise ASMRs trajectories suggest gradual declines from current levels: EC from approximately the upper 0.7–0.8 per 100000 range toward lower values, SC from around 0.5–0.6 toward 0.3–0.4, LC from roughly 0.3–0.4 toward 0.2–0.3, PC from near 2.0–2.5 toward 1.5–2.0, and CRC from about 1.5–2.0 toward 1.0–1.5 per 100000, with variation by region and sex. Consistent with these trends, the aggregate ASMR for smoking-related digestive cancers in older adults is projected to decline modestly but steadily through 2036, while the corresponding death counts and DALYs rise due to demographic momentum (Supplementary file Figure S7).

## DISCUSSION

This study provides a comprehensive, age-focused appraisal of smoking-attributable burdens for EC, SC, LC, PC, and CRC among adults aged ≥60 years, integrating decomposition and SDI correlation analyses across 204 countries using GBD 2021 estimates, and describing how population growth, aging, and epidemiological change were related to the observed trends from 1990 to 2021^24^. Despite sustained declines in age-standardized ASMR and ASDR across cancer sites, absolute deaths and DALYs rose. In our decomposition analyses, most of this increase was accounted for by population growth (41–43%), with smaller contributions from population aging (5–15%) and partial offset from declining age-specific rates, suggesting epidemiological gains that may not have been sufficient to balance demographic pressures in older cohorts^16^. Pronounced heterogeneity by sex, age, region, and development level was mechanistically coherent: the largest absolute burdens accrued to older men in East and South Asia, Western Europe, and Sub-Saharan Africa, while rate declines were steepest in high-SDI settings, consistent with earlier and stronger tobacco control and greater screening and treatment capacity.

Sex disparities remained marked, with male-to-female ratios frequently exceeding 3:1, consistent with historically higher smoking prevalence and intensity, earlier initiation, slower cessation, and more frequent co-exposures (e.g. alcohol and certain occupational agents) reported among men, which may together increase cumulative carcinogen exposure^25^. Biological factors may accentuate these differences: sex-specific variation in nicotine metabolism, steroid hormone signaling, and immune function could modify susceptibility, while higher levels of tobacco-derived nitrosamines and polycyclic aromatic hydrocarbons could plausibly contribute to greater DNA adduct formation and chromosomal instability along the gastrointestinal tract in men. In EC, chronic exposure to smoking and alcohol in the presence of ALDH2 polymorphisms that impair acetaldehyde metabolism has been associated with a higher risk, particularly in East Asian men. In SC, smoking has been reported to act synergistically with *Helicobacter pylori*-induced chronic gastritis and atrophy. In LC, smoking may augment oxidative stress and fibrogenesis on a background of HBV/HCV infection. In PC, tobacco exposure has been implicated in promoting KRAS-driven oncogenesis and stellate cell activation. In CRC, smoking has been linked to changes in the microbiome and prostaglandin signaling while intersecting with Wnt/β-catenin pathways, with these patterns reported to be more pronounced with higher cumulative exposure in late life^26-30^. Encouragingly, the gender gap in age-standardized rates narrowed modestly in high-SDI regions, paralleling earlier declines in male smoking and broader uptake of cessation services, and is consistent with, but does not prove, a potential impact of tobacco control policies on these trends.

Age gradients were consistent with carcinogenic biology and clinical realities in later life. The steepest increases in absolute deaths and DALYs occurred in ages 75–89 years, which may reflect latency from midlife exposures and cohort aging, while small late-life reversals or plateaus in ASMR/ASDR for CRC and LC among the very old may be related to limits in screening eligibility, diagnostic intensity, and treatment tolerance amid frailty, multimorbidity, and polypharmacy^30^. The oncogenic impact of tobacco may be amplified in older tissues through age-related immune senescence, diminished DNA repair, and cumulative oxidative injury. This process may be further influenced by age-related chronic inflammatory remodeling, which has been associated with carcinogenesis through distinct pathways in specific organs: hepatic fibrosis in LC, gastric atrophy in SC, and desmoplastic stroma in PC. These patterns underscore the relevance of geriatric-oncology pathways, incorporating comprehensive geriatric assessment, toxicity-adaptive regimens, and individualized, life-expectancy-aligned screening decisions to preserve benefit–harm balance in older adults^30^.

Regional and SDI-stratified analyses revealed development-linked ‘tipping points’. In high-SDI regions, strong epidemiological gains appeared sufficient to offset demographic pressures. These favorable trends are consistent with the long-term implementation of comprehensive tobacco control measures, encompassing taxation, standardized packaging, smoke-free laws, and advertising bans, integrated with accessible cessation support. When complemented by mature screening and multidisciplinary care, these strategies have together reduced mortality by 20–40% in trials and real-world programs^24,31,32^. By contrast, low- and low-middle SDI regions experienced escalating burdens in the context of rapid population growth and only modest rate improvements, coinciding with challenges such as limited policy enforcement, under-resourced primary care, competing priorities such as infectious diseases and undernutrition, and slow scale-up of infection control for HBV/HCV and *H. pylori* ^33,34^. Country-level correlations between SDI and absolute burden were positive (ρ=0.20–0.48), peaking at SDI=0.70–0.85, consistent with an ‘epidemiological paradox’ in which early development elevates burden through aging and longer survival, followed by declines at the highest SDI levels as prevention and care mature; regionally, an ‘M-shaped’ relationship suggested heterogeneity in age structures, health systems, and historical exposures, with declines emerging past high development thresholds^16^. Site-specific geographies were mechanistically concordant: EC concentrated where smoking, alcohol, and ALDH2 variants co-occur; SC mirrored the intersection of smoking and *H. pylori* with large epidemiological declines in middle-SDI East Asia after eradication programs; LC clustered with HBV/HCV endemicity and smoking, with vaccination and antiviral therapy that may help to mitigate this synergistic risk; PC and CRC peaked in high-SDI regions but showed the strongest rate-driven declines where screening and integrated oncology are entrenched.

These findings suggest policy priorities but cannot prove cause and effect. Even if rates decline, the overall burden remains high. In high-SDI settings, trends may warrant sustaining adaptable tobacco control frameworks for aging populations, such as integrating cessation support with chronic disease management and exploring risk-appropriate screening pathways for older adults^31,32^. Middle-SDI regions could benefit from strengthening policy enforcement and primary-care-based cessation interventions, while identifying cost-effective opportunities to expand screening and infection control. In low and low-middle SDI settings, prioritizing foundational tobacco control measures alongside investments in diagnostic infrastructure and cancer registration systems may help guide future scale-up, potentially supported by international partnerships^33,34^. Given that population growth accounted for the largest share of the increase in case counts, our descriptive analysis suggests primary prevention targeting younger cohorts could be a logical focus for future intervention research, though effectiveness and equity implications require further investigation.

### Limitations

This analysis has several limitations. First, as a secondary analysis of aggregated, modelled GBD estimates, our findings are subject to measurement and modeling uncertainty (especially in data-scarce settings) and should be interpreted as ecological associations rather than individual-level causal effects. Accordingly, causal inferences cannot be established, and there is potential residual confounding from incompletely captured determinants (e.g. air pollution, occupational exposures, diet/metabolic risks, healthcare access, screening, diagnostic and treatment changes, and broader socioeconomic shifts). In addition, smoking-attributable burden may reflect co-exposures and interactions with other behavioral and environmental factors (and omission of protective factors such as diet quality, physical activity, and aspirin), which we could not explicitly model, potentially confounding SDI relationships. Our BAPC projections assume a relatively smooth continuation of past age–period–cohort patterns and therefore may not capture future shocks or structural breaks (e.g. pandemics, policy reversals, economic disruptions, conflicts, or rapid diffusion of heated tobacco and e-cigarettes)^35,36^, with uncertainty increasing over longer horizons. Moreover, projections focused on adults aged ≥60 years may not be generalizable to younger adults, and EAPC summarizes trends under a log-linear assumption that may mask non-linear temporal changes. Nonetheless, convergent patterns across decomposition, SDI correlations, and projections suggest a coherent picture in aging societies: rising absolute burdens occur alongside falling age-specific risks. These descriptive findings may help inform sustained, equitable tobacco control and geriatric-appropriate prevention, detection, and treatment strategies.

## CONCLUSIONS

This study assessed the global burden of smoking-attributable gastrointestinal cancers from 1990 to 2021 and projected trends to 2036. Results indicate declining age-standardized rates, potentially associated with reduced smoking prevalence and advances in prevention, screening, and treatment. Nevertheless, males and older adults continue to bear disproportionately higher burdens, while rates among females have remained relatively stable. Reducing tobacco consumption and ensuring equitable cancer care could be supported by strategic resource allocation and robust policy implementation, particularly in rapidly developing countries. Simultaneously, context-specific localized interventions may offer opportunities to address the burden of preventable smoking-related gastrointestinal cancers in globally aging populations.

## Supplementary Material



## Data Availability

The data supporting this research are available from the following sources: Institute for Health Metrics and Evaluation (http://ghdx.healthdata.org).

## References

[1] GBD 2021 Risk Factors Collaborators. Global burden and strength of evidence for 88 risk factors in 204 countries and 811 subnational locations, 1990-2021: A systematic analysis for the Global Burden of Disease Study 2021. Lancet. 2024;403(10440):2162-2203. doi:10.1016/S0140-6736(24)00933-4 38762324 PMC11120204

[2] Keum N, Giovannucci E. Global burden of colorectal cancer: Emerging trends, risk factors and prevention strategies. Nat Rev Gastroenterol Hepatol. 2019;16(12):713-732. doi:10.1038/s41575-019-0189-8 31455888

[3] Vater L, Trikannad Ashwini Kumar A, Sehgal N, et al. Tobacco use, knowledge of harms, and cessation support among patients with non-tobacco related cancers. J Clin Oncol. 2021;39(15_suppl):e24029. doi:10.1200/JCO.2021.39.15_suppl.e24029

[4] Secretan B, Straif K, Baan R, et al. A review of human carcinogens--Part E: Tobacco, areca nut, alcohol, coal smoke, and salted fish. Lancet Oncol. 2009;10(11):1033-1034. doi:10.1016/s1470-2045(09)70326-2 19891056

[5] Kennedy BK, Berger SL, Brunet A, et al. Geroscience: Linking aging to chronic disease. Cell. 2014;159(4):709-713. doi:10.1016/j.cell.2014.10.039 25417146 PMC4852871

[6] Wildiers H, Heeren P, Puts M, et al. International Society of Geriatric Oncology consensus on geriatric assessment in older patients with cancer. J Clin Oncol. 2014;32(24):2595-2603. doi:10.1200/JCO.2013.54.8347 25071125 PMC4876338

[7] Lee K, Egbe CO, Bianco E, Arora M. The 20th anniversary of the WHO Framework Convention on Tobacco Control: Hard won progress amid evolving challenges. Lancet. 2023;402(10402):592-594. doi:10.1016/S0140-6736(23)01080-2 37263281

[8] Bilano V, Gilmour S, Moffiet T, et al. Global trends and projections for tobacco use, 1990-2025: An analysis of smoking indicators from the WHO Comprehensive Information Systems for Tobacco Control. Lancet. 2015;385(9972):966-976. doi:10.1016/s0140-6736(15)60264-1 25784347

[9] Bray F, Laversanne M, Weiderpass E, Soerjomataram I. The ever-increasing importance of cancer as a leading cause of premature death worldwide. Cancer. 2021;127(16):3029-3030. doi:10.1002/cncr.33587 34086348

[10] Islami F, Marlow EC, Thomson B, et al. Proportion and number of cancer cases and deaths attributable to potentially modifiable risk factors in the United States, 2019. CA Cancer J Clin. 2024;74(5):405-432. doi:10.3322/caac.21858 38990124

[11] Arnold M, Abnet CC, Neale RE, et al. Global burden of 5 major types of gastrointestinal cancer. Gastroenterology. 2020;159(1):335-349. doi:10.1053/j.gastro.2020.02.068 32247694 PMC8630546

[12] Cheng X, Yang Y, Schwebel DC. Population ageing and mortality during 1990-2017: A global decomposition analysis. PLoS Med. 2020;17(6):e1003138. doi:10.1371/journal.pmed.1003138 32511229 PMC7279585

[13] Global Burden of Disease 2019 Cancer Collaboration, Kocarnik JM, Compton K, et al. Cancer incidence, mortality, years of life lost, years lived with disability, and disability-adjusted life years for 29 cancer groups from 2010 to 2019: A systematic analysis for the Global Burden of Disease Study 2019. JAMA Oncol. 2022;8(3):420-444. doi:10.1001/jamaoncol.2021.6987 34967848 PMC8719276

[14] Luo Z, Huang Y, Ye R, Yin M. Global burden and gender disparities in head and neck cancers among adults aged 40-64, 1990-2021: A systematic analysis from the Global Burden of Disease Study 2021. Cancer Rep (Hoboken). 2025;8(8):e70287. doi:10.1002/cnr2.70287 40832837 PMC12365661

[15] Stevens GA, Alkema L, Black RE, et al. Guidelines for accurate and transparent health estimates reporting: The GATHER statement. Lancet. 2016;388(10062):e19-e23. doi:10.1016/S0140-6736(16)30388-9 27371184

[16] Ren Y, Xu R, Wang Y, Su L, Su J. Global, regional, and national burden of ovarian cancer in women aged 45 + from 1990 to 2021 and projections for 2050: A systematic analysis based on the 2021 global burden of disease study. J Cancer Res Clin Oncol. 2025;151(8):225. doi:10.1007/s00432-025-06277-9 40751826 PMC12317933

[17] Riebler A, Held L. Projecting the future burden of cancer: Bayesian age-period-cohort analysis with integrated nested Laplace approximations. Biom J. 2017;59(3):531-549. doi:10.1002/bimj.201500263 28139001

[18] Knoll M, Furkel J, Debus J, Abdollahi A, Karch A, Stock C. An R package for an integrated evaluation of statistical approaches to cancer incidence projection. BMC Med Res Methodol. 2020;20(1):257. doi:10.1186/s12874-020-01133-5 33059585 PMC7559591

[19] The R Foundation. The R Project for Statistical Computing; 2021. Accessed December 26, 2025. https://www.R-project.org/

[20] StataCorp. Stata Statistical Software: Release 18. Accessed December 26, 2025. https://www.stata.com/stata18/

[21] Environmental Systems Research Institute. ArcGIS Pro. Version 3.0. Accessed December 26, 2025. https://www.esri.com/en-us/arcgis/products/arcgis-pro/

[22] QGIS Development Team. QGIS Geographic Information System. Version 3.28. Accessed December 26, 2025. https://qgis.org/

[23] Kim HJ, Fay MP, Feuer EJ, Midthune DN. Permutation tests for joinpoint regression with applications to cancer rates. Stat Med. 2000;19(3):335-351. doi:10.1002/(sici)1097-0258(20000215)19:3<335::aid-sim336>3.0.co;2-z 10649300

[24] Korthauer K, Kimes PK, Duvallet C, et al. A practical guide to methods controlling false discoveries in computational biology. Genome Biol. 2019;20(1):118. doi:10.1186/s13059-019-1716-1 31164141 PMC6547503

[25] Fountoukidis G, Schiza A, Smith D, et al. Effect of alcohol consumption on oncological treatment effectiveness and toxicity in patients with cancer: A systematic review and meta-analysis. BMC cancer. 2025;25(1):246. doi:10.1186/s12885-025-13694-z 39939877 PMC11823036

[26] Ford AC, Yuan Y, Moayyedi P. Helicobacter pylori eradication therapy to prevent gastric cancer: Systematic review and meta-analysis. Gut. 2020;69(12):2113-2121. doi:10.1136/gutjnl-2020-320839 32205420

[27] Chen J, Qin Z, Wang X, et al. Regional and gender disparities in tobacco-related esophageal cancer: Insights from the Global Burden of Disease study 1990-2021. Tob Induc Dis. 2025;23:96. doi:10.18332/tid/205670 PMC12278272 40692961

[28] Dominguez A, Avellon A, Hernando V, et al. Hepatitis B virus-related cirrhosis and hepatocellular carcinoma hospital discharge rates from 2005 to 2021 in SpaIn: Impact of universal vaccination. Vaccines (Basel). 2024;12(11):1254. doi:10.3390/vaccines12111254 39591157 PMC11598889

[29] Akter S, Rahman MM, Rouyard T, Aktar S, Nsashiyi RS, Nakamura R. A systematic review and network meta-analysis of population-level interventions to tackle smoking behaviour. Nat Hum Behav. 2024;8(12):2367-2391. doi:10.1038/s41562-024-02002-7 39375543 PMC11659173

[30] Yu W, Zhou D, Meng F, et al. The global, regional burden of pancreatic cancer and its attributable risk factors from 1990 to 2021. BMC cancer. 2025;25(1):186. doi:10.1186/s12885-025-13471-y 39891086 PMC11786447

[31] VanFrank B, Presley-Cantrell L. A comprehensive approach to increase adult tobacco cessation. JAMA. 2021;325(3):232-233. doi:10.1001/jama.2020.23608 33464294 PMC11000816

[32] Hamoud J, Hanewinkel R, Andreas S, et al. A systematic review investigating the impact of dual use of e-cigarettes and conventional cigarettes on smoking cessation. ERJ Open Res. 2025;11(3):00902-02024. doi:10.1183/23120541.00902-2024 40337339 PMC12053992

[33] Paraje G, Munoz MF, Wu D, Jha P. Tobacco kills: Stronger excise taxes would save lives. BMJ (Clinical research edition). 2025;390:e085608. doi:10.1136/bmj-2025-085608 PMC12444781 40940083

[34] Jha P, Peto R. Global effects of smoking, of quitting, and of taxing tobacco. N Engl J Med. 2014;370(1):60-68. doi:10.1056/NEJMra1308383 24382066

[35] Hartmann-Boyce J, Livingstone-Banks J, Ordonez-Mena JM, et al. Behavioural interventions for smoking cessation: an overview and network meta-analysis. Cochrane Database Syst Rev. 2021;1(1):CD013229. doi:10.1002/14651858.CD013229.pub2 33411338 PMC11354481

[36] Skriver C, Maltesen T, Dehlendorff C, et al. Long-term aspirin use and cancer risk: A 20-year cohort study. J Natl Cancer Inst. 2024;116(4):530-538. doi:10.1093/jnci/djad231 37966913

