# Supplementary material for: Global, regional, and national burden of smoking-attributable digestive cancers in adults aged ≥60 years, 1990–2021, with projections to 2036: A secondary dataset analysis of Global Burden of Disease (GBD) 2021
Source: Tob Induc Dis. 2026 Feb 17;24:10.18332/tid/216110. doi: 10.18332/tid/216110 (PMC12915483; doi:10.18332/tid/216110)
Supplement: Supplementary file 1 [file TID-24-25-s1.pdf]

Supplementary Table S1 Global and regional numbers and age-standardized DALY rates for esophageal cancer attributable to smoking in adults aged ≥60 years, with EAPC from 1990 to 2021, based on GBD 2021.

location	1990		2021		EAPC（95%CI）
	Number（95%UI）	ASR(per 100,000，95%UI)	Number（95%UI）	ASR(per 100,000，95%UI)	
Andean Latin America	901 (640 to 1222)	38.37 (27.2 to 52.18)	1544 (1021 to 2239)	21.6 (14.27 to 31.32)	-1.81 (-1.94 to -1.68)
Australasia	6596 (4885 to 8459)	210.24 (155.44 to 270.15)	7049 (4639 to 9906)	99.01 (65.45 to 138.62)	-2.59 (-2.66 to -2.51)
Caribbean	4705 (3508 to 6025)	147.1 (109.42 to 188.72)	7899 (5689 to 10596)	117.83 (84.91 to 158.05)	-0.47 (-0.57 to -0.37)
Central Asia	23955 (18551 to 29529)	412.22 (318.14 to 509.35)	15722 (11985 to 19661)	158.72 (120.79 to 198.83)	-2.97 (-3.05 to -2.89)
Central Europe	29331 (23912 to 34575)	145.69 (118.45 to 172.14)	39214 (30453 to 48442)	132.43 (103.04 to 163.4)	-0.45 (-0.57 to -0.33)
Central Latin America	7110 (5427 to 8992)	75.6 (57.5 to 95.91)	8221 (5977 to 10635)	26.78 (19.45 to 34.69)	-3.58 (-3.69 to -3.46)
Central Sub-Saharan Africa	3466 (2279 to 5021)	127.27 (83.56 to 184.71)	5539 (3666 to 8005)	88.75 (58.88 to 128.47)	-1.23 (-1.52 to -0.94)
East Asia	1290793 (975614 to 1630910)	1212.26 (915.03 to 1531.36)	2259249 (1640054 to 3001787)	804.75 (584.68 to 1068.19)	-1.39 (-1.53 to -1.25)
Eastern Europe	72007 (58742 to 84367)	186.21 (151.55 to 218.7)	70838 (56467 to 85597)	143.54 (114.33 to 173.61)	-1.05 (-1.17 to -0.93)
Eastern Sub-Saharan Africa	16236 (11949 to 20933)	186.4 (136.97 to 240.53)	23913 (17595 to 31789)	126 (92.5 to 167.33)	-1.54 (-1.66 to -1.43)
Global	2074970 (1644762 to 2503230)	417.95 (330.88 to 504.46)	3268824 (2472935 to 4131854)	298.69 (225.9 to 377.57)	-1.18 (-1.25 to -1.1)
High-income Asia Pacific	90901 (74876 to 106487)	354.41 (291.43 to 415.99)	113284 (84855 to 141541)	187.88 (142.04 to 233.8)	-2.25 (-2.42 to -2.08)

High-income North America	122949 (98259 to 145723)	267.26 (213.87 to 316.37)	188637 (141710 to 234335)	213.43 (160.54 to 264.84)	-0.86 (-1.04 to -0.69)
High-middle SDI	691268 (541344 to 854936)	537.92 (420.6 to 665.72)	1180626 (866351 to 1584921)	457.59 (335.63 to 613.91)	-0.55 (-0.65 to -0.45)
High SDI	442622 (361579 to 516478)	307.13 (250.9 to 358.37)	573943 (444717 to 702323)	209.83 (163.28 to 255.97)	-1.39 (-1.51 to -1.27)
Low-middle SDI	91580 (71073 to 113152)	128.44 (99.3 to 158.87)	151620 (116526 to 187906)	87.41 (67.14 to 108.46)	-1.38 (-1.46 to -1.29)
Low SDI	27661 (20776 to 34636)	104.5 (78.24 to 131.17)	41329 (30717 to 52965)	71.41 (52.99 to 91.7)	-1.48 (-1.58 to -1.39)
Middle SDI	820956 (628944 to 1038516)	659.37 (505.11 to 833.09)	1320122 (951792 to 1752863)	397.94 (287.01 to 527.9)	-1.68 (-1.77 to -1.6)
North Africa and Middle East	18079 (13615 to 22766)	92.24 (69.26 to 116.58)	38793 (28963 to 49303)	76.32 (56.75 to 97.29)	-0.72 (-0.77 to -0.66)
Oceania	198 (131 to 291)	57.51 (37.99 to 83.81)	353 (244 to 505)	41.81 (28.82 to 59.97)	-1.16 (-1.22 to -1.1)
South Asia	75181 (57025 to 95548)	113.56 (85.64 to 144.78)	128005 (95187 to 163317)	71.39 (53.12 to 91.27)	-1.8 (-1.93 to -1.68)
Southeast Asia	30848 (23279 to 40747)	103.61 (78.16 to 136.76)	62704 (47943 to 79938)	77.77 (59.37 to 99.31)	-1.14 (-1.21 to -1.07)
Southern Latin America	16638 (12084 to 21655)	277.55 (200.83 to 362.28)	13980 (9784 to 18683)	124.59 (87.34 to 166.31)	-2.62 (-2.91 to -2.33)
Southern Sub-Saharan Africa	11243 (8310 to 14619)	354.05 (260.46 to 461.61)	16129 (11740 to 21124)	227.15 (164.14 to 299.13)	-1.97 (-2.42 to -1.51)
Tropical Latin America	41768 (32453 to 51215)	383.69 (297.03 to 472.69)	54183 (39608 to 70244)	166.96 (121.77 to 216.76)	-2.86 (-2.95 to -2.77)
Western Europe	209601 (169291 to 246350)	276.83 (223.9 to 325.07)	205923 (155445 to 256060)	174.5 (132.85 to 215.93)	-1.53 (-1.59 to -1.46)
Western Sub-Saharan Africa	2464 (1751 to 3378)	23.38 (16.56 to 32.12)	7647 (5389 to 10275)	33.86 (23.82 to 45.65)	1.68 (1.49 to 1.87)

DALYs: disability-adjusted life years; ASR: age-standardized rate; UI: uncertainty intervals; CI: confidence interval; SDI: socio-demographic index; EAPC: estimated annual percentage change.

Supplementary Table S2 Global and regional numbers and age-standardized death rates for esophageal cancer attributable to smoking in adults aged ≥60 years, with EAPC from 1990 to 2021, based on GBD 2021.

location	1990		2021		EAPC（95%CI）
	Number（95%UI）	ASR(per 100,000，95%UI)	Number（95%UI）	ASR(per 100,000，95%UI)	
Andean Latin America	46 (32 to 63)	2.02 (1.41 to 2.78)	83 (55 to 121)	1.18 (0.78 to 1.72)	-1.7 (-1.82 to -1.57)
Australasia	324 (237 to 420)	10.41 (7.6 to 13.56)	394 (255 to 563)	5.34 (3.47 to 7.59)	-2.33 (-2.42 to -2.24)
Caribbean	241 (177 to 311)	7.76 (5.69 to 10.06)	393 (280 to 531)	5.84 (4.16 to 7.88)	-0.7 (-0.79 to -0.6)
Central Asia	1071 (824 to 1325)	19.23 (14.72 to 23.84)	711 (540 to 891)	7.71 (5.85 to 9.7)	-2.77 (-2.86 to -2.67)
Central Europe	1328 (1071 to 1580)	6.85 (5.5 to 8.18)	1809 (1387 to 2255)	6.02 (4.62 to 7.49)	-0.57 (-0.68 to -0.45)
Central Latin America	364 (275 to 463)	4.1 (3.08 to 5.24)	425 (307 to 554)	1.42 (1.02 to 1.85)	-3.64 (-3.76 to -3.53)
Central Sub-Saharan Africa	146 (96 to 211)	5.86 (3.84 to 8.52)	235 (156 to 340)	4.1 (2.72 to 5.95)	-1.22 (-1.52 to -0.92)
East Asia	58576 (44262 to 73874)	59.97 (45.18 to 75.68)	114589 (83431 to 151718)	42.42 (30.93 to 56.05)	-1.21 (-1.38 to -1.04)
Eastern Europe	3055 (2478 to 3599)	8.17 (6.6 to 9.66)	3048 (2419 to 3708)	6.26 (4.96 to 7.62)	-1.04 (-1.15 to -0.93)
Eastern Sub-Saharan Africa	719 (529 to 928)	9.03 (6.62 to 11.68)	1073 (787 to 1423)	6.1 (4.46 to 8.08)	-1.55 (-1.66 to -1.43)
Global	95709 (75653 to 115530)	20.05 (15.82 to 24.24)	165477 (124966 to 209406)	15.4 (11.62 to 19.49)	-0.98 (-1.07 to -0.88)
High-income Asia Pacific	4349 (3568 to 5120)	17.43 (14.26 to 20.59)	6527 (4823 to 8206)	9.89 (7.39 to 12.36)	-2.02 (-2.15 to -1.9)

High-income North America	5904 (4661 to 7067)	12.62 (9.97 to 15.09)	9559 (7039 to 12051)	10.7 (7.89 to 13.47)	-0.67 (-0.85 to -0.49)
High-middle SDI	31655 (24690 to 39221)	25.67 (19.97 to 31.85)	59429 (43557 to 79544)	23.33 (17.09 to 31.2)	-0.38 (-0.5 to -0.26)
High SDI	21608 (17494 to 25413)	14.92 (12.07 to 17.56)	30630 (23374 to 37883)	10.73 (8.23 to 13.21)	-1.23 (-1.36 to -1.11)
Low-middle SDI	4145 (3200 to 5131)	6.28 (4.82 to 7.79)	7124 (5471 to 8841)	4.35 (3.34 to 5.41)	-1.3 (-1.38 to -1.22)
Low SDI	1231 (922 to 1545)	5.08 (3.78 to 6.4)	1886 (1398 to 2426)	3.51 (2.6 to 4.53)	-1.43 (-1.52 to -1.33)
Middle SDI	37028 (28396 to 46760)	31.7 (24.29 to 39.97)	66351 (47935 to 87902)	20.89 (15.1 to 27.63)	-1.45 (-1.56 to -1.35)
North Africa and Middle East	834 (626 to 1054)	4.63 (3.45 to 5.88)	1933 (1434 to 2468)	4.11 (3.03 to 5.27)	-0.43 (-0.49 to -0.38)
Oceania	8 (6 to 12)	2.72 (1.8 to 3.94)	15 (11 to 22)	1.96 (1.35 to 2.83)	-1.19 (-1.25 to -1.12)
South Asia	3371 (2539 to 4300)	5.54 (4.14 to 7.11)	6079 (4530 to 7777)	3.61 (2.69 to 4.64)	-1.67 (-1.79 to -1.55)
Southeast Asia	1398 (1054 to 1842)	5.04 (3.79 to 6.63)	2892 (2205 to 3694)	3.86 (2.93 to 4.95)	-1.08 (-1.15 to -1.01)
Southern Latin America	784 (560 to 1031)	13.4 (9.5 to 17.7)	700 (481 to 947)	6.16 (4.24 to 8.33)	-2.49 (-2.79 to -2.2)
Southern Sub-Saharan Africa	540 (395 to 706)	18.22 (13.24 to 23.93)	721 (519 to 952)	10.88 (7.75 to 14.49)	-2.22 (-2.68 to -1.76)
Tropical Latin America	1956 (1509 to 2418)	19.15 (14.69 to 23.85)	2615 (1883 to 3418)	8.22 (5.9 to 10.77)	-2.84 (-2.92 to -2.77)
Western Europe	10585 (8472 to 12544)	13.75 (11.01 to 16.28)	11341 (8401 to 14255)	8.92 (6.68 to 11.14)	-1.45 (-1.52 to -1.38)
Western Sub-Saharan Africa	110 (78 to 151)	1.11 (0.78 to 1.53)	335 (235 to 452)	1.58 (1.1 to 2.14)	1.63 (1.44 to 1.82)

ASR: age-standardized rate; UI: uncertainty intervals; CI: confidence interval; SDI: socio-demographic index; EAPC: estimated annual percentage change.

Supplementary Table S3 Global and regional numbers and age-standardized DALY rates for colorectal cancer attributable to smoking in adults aged ⩾60 years, with EAPC from 1990 to 2021, based on GBD 2021.

location	1990		2021		EAPC（95%CI）
	Number（95%UI）	ASR(per 100,000，95%UI)	Number（95%UI）	ASR(per 100,000，95%UI)	
Andean Latin America	673 (393 to 1002)	28.45 (16.57 to 42.38)	2117 (1174 to 3353)	29.46 (16.33 to 46.69)	0.31 (0.21 to 0.4)
Australasia	4484 (2734 to 6411)	143.03 (87.15 to 204.61)	3523 (2007 to 5482)	49.83 (28.48 to 77.26)	-3.76 (-3.89 to -3.63)
Caribbean	2188 (1307 to 3157)	67.9 (40.53 to 98.06)	4247 (2503 to 6256)	63.36 (37.36 to 93.32)	-0.16 (-0.24 to -0.08)
Central Asia	2444 (1536 to 3405)	40.84 (25.62 to 56.94)	3656 (2255 to 5144)	35.98 (22.18 to 50.72)	0.19 (0.02 to 0.36)
Central Europe	28146 (17812 to 38896)	140.15 (88.49 to 194.1)	41585 (25727 to 58745)	139.39 (86.35 to 196.75)	-0.05 (-0.17 to 0.08)
Central Latin America	3275 (2014 to 4614)	34.28 (21.06 to 48.39)	9748 (5838 to 13968)	31.45 (18.8 to 45.09)	-0.38 (-0.45 to -0.31)
Central Sub-Saharan Africa	503 (281 to 780)	19.09 (10.61 to 29.86)	1026 (575 to 1630)	16.79 (9.41 to 26.65)	-0.25 (-0.59 to 0.1)
East Asia	105305 (66790 to 148331)	100.57 (63.46 to 141.85)	262252 (154492 to 390466)	93.42 (55.04 to 139.18)	-0.08 (-0.19 to 0.04)
Eastern Europe	34307 (21820 to 47266)	89.93 (57.09 to 123.98)	46347 (28885 to 64953)	94.84 (59.07 to 133.05)	-0.07 (-0.36 to 0.21)
Eastern Sub-Saharan Africa	2772 (1642 to 4060)	32.17 (19.08 to 47.11)	4728 (2775 to 7025)	25.95 (15.19 to 38.51)	-0.76 (-0.88 to -0.64)
Global	460408 (291227 to 625129)	94.11 (59.33 to 128.09)	727595 (451515 to 1021401)	66.51 (41.24 to 93.47)	-1.19 (-1.23 to -1.16)
High-income Asia Pacific	32689 (20733 to 44441)	127.78 (80.87 to 174)	42509 (25369 to 61691)	72.39 (43.67 to 104.46)	-2.1 (-2.19 to -2.02)

High-income North America	61147 (37419 to 85797)	131.99 (80.81 to 184.93)	55086 (32397 to 80978)	62.51 (36.82 to 91.79)	-2.66 (-2.78 to -2.55)
High-middle SDI	137058 (87305 to 187441)	106.79 (67.85 to 146.26)	243978 (150076 to 343033)	94.51 (58.11 to 132.86)	-0.41 (-0.49 to -0.33)
High SDI	210258 (132660 to 290550)	145.48 (91.83 to 201.02)	210056 (127154 to 300101)	77.07 (46.8 to 109.75)	-2.22 (-2.28 to -2.16)
Low-middle SDI	22907 (14087 to 32421)	31.85 (19.54 to 45.17)	51837 (32243 to 73566)	29.68 (18.43 to 42.15)	-0.29 (-0.34 to -0.24)
Low SDI	5963 (3587 to 8604)	22.55 (13.53 to 32.54)	10040 (6050 to 14397)	17.65 (10.63 to 25.31)	-0.89 (-0.97 to -0.8)
Middle SDI	83556 (53106 to 114858)	68.32 (43.37 to 94.02)	210727 (130215 to 301244)	63.1 (38.95 to 90.36)	-0.22 (-0.29 to -0.15)
North Africa and Middle East	8416 (4951 to 12406)	42.49 (24.97 to 62.79)	21093 (12567 to 30476)	40.21 (23.86 to 58.15)	-0.14 (-0.25 to -0.04)
Oceania	102 (59 to 150)	29.33 (17.1 to 43.39)	195 (115 to 287)	23 (13.49 to 33.87)	-0.89 (-0.97 to -0.8)
South Asia	16931 (10220 to 24363)	25.27 (15.18 to 36.5)	34506 (21177 to 49601)	19.01 (11.66 to 27.39)	-1.1 (-1.21 to -1)
Southeast Asia	20706 (12823 to 29354)	70.36 (43.52 to 99.78)	64017 (39196 to 93576)	79.61 (48.63 to 116.34)	0.28 (0.18 to 0.38)
Southern Latin America	7030 (4192 to 10375)	116.51 (69.34 to 172.23)	10488 (6095 to 15625)	93.97 (54.64 to 139.83)	-0.49 (-0.67 to -0.31)
Southern Sub-Saharan Africa	1382 (842 to 2040)	43.67 (26.56 to 64.54)	2678 (1604 to 3918)	37.88 (22.59 to 55.58)	-0.47 (-0.69 to -0.24)
Tropical Latin America	7614 (4599 to 11007)	69.97 (42.08 to 101.64)	18676 (11173 to 27240)	57.42 (34.27 to 83.85)	-0.78 (-0.87 to -0.69)
Western Europe	119351 (74667 to 166233)	156.37 (97.99 to 217.66)	97249 (58052 to 139864)	82.77 (49.77 to 118.37)	-2.1 (-2.14 to -2.05)
Western Sub-Saharan Africa	944 (547 to 1411)	9.21 (5.33 to 13.74)	1869 (1066 to 2713)	8.59 (4.9 to 12.5)	-0.06 (-0.14 to 0.01)

DALYs: disability-adjusted life years; ASR: age-standardized rate; UI: uncertainty intervals; CI: confidence interval; SDI: socio-demographic index; EAPC: estimated annual percentage change.

Supplementary Table S4 Global and regional numbers and age-standardized death rates for colorectal cancer attributable to smoking in adults aged ≥60 years, with EAPC from 1990 to 2021, based on GBD 2021.

location	1990		2021		EAPC（95%CI）
	Number（95%UI）	ASR(per 100,000, 95%UI)	Number（95%UI）	ASR(per 100,000, 95%UI)	
Andean Latin America	33 (19 to 49)	1.44 (0.83 to 2.16)	106 (59 to 170)	1.5 (0.83 to 2.4)	0.34 (0.24 to 0.43)
Australasia	207 (125 to 299)	6.64 (3.99 to 9.59)	182 (102 to 290)	2.48 (1.39 to 3.93)	-3.48 (-3.59 to -3.36)
Caribbean	106 (63 to 153)	3.36 (1.99 to 4.87)	207 (120 to 308)	3.08 (1.79 to 4.58)	-0.23 (-0.31 to -0.15)
Central Asia	102 (64 to 143)	1.78 (1.11 to 2.48)	158 (98 to 223)	1.66 (1.02 to 2.35)	0.48 (0.28 to 0.67)
Central Europe	1262 (793 to 1752)	6.48 (4.06 to 9.03)	1935 (1189 to 2748)	6.39 (3.93 to 9.07)	-0.08 (-0.22 to 0.05)
Central Latin America	160 (98 to 226)	1.76 (1.08 to 2.5)	460 (273 to 663)	1.52 (0.9 to 2.19)	-0.57 (-0.64 to -0.5)
Central Sub-Saharan Africa	22 (12 to 34)	0.91 (0.5 to 1.43)	44 (25 to 70)	0.79 (0.44 to 1.26)	-0.29 (-0.65 to 0.06)
East Asia	4763 (3007 to 6720)	5.04 (3.15 to 7.12)	12662 (7488 to 18833)	4.69 (2.77 to 6.98)	-0.1 (-0.2 to -0.01)
Eastern Europe	1473 (934 to 2034)	3.98 (2.51 to 5.5)	2034 (1258 to 2863)	4.23 (2.61 to 5.96)	-0.02 (-0.32 to 0.28)
Eastern Sub-Saharan Africa	124 (73 to 181)	1.58 (0.94 to 2.31)	222 (130 to 328)	1.34 (0.78 to 1.98)	-0.58 (-0.7 to -0.46)
Global	21494 (13494 to 29317)	4.62 (2.89 to 6.33)	35036 (21566 to 49366)	3.26 (2.01 to 4.61)	-1.21 (-1.24 to -1.17)
High-income Asia Pacific	1506 (945 to 2060)	6.09 (3.81 to 8.35)	2278 (1339 to 3328)	3.49 (2.09 to 5.07)	-2.06 (-2.15 to -1.98)

High-income North America	2907 (1756 to 4133)	6.18 (3.73 to 8.77)	2645 (1525 to 3956)	2.96 (1.71 to 4.42)	-2.62 (-2.74 to -2.5)
High-middle SDI	6226 (3941 to 8539)	5.09 (3.21 to 6.99)	11554 (7044 to 16308)	4.53 (2.76 to 6.39)	-0.39 (-0.48 to -0.3)
High SDI	10167 (6330 to 14140)	7.01 (4.36 to 9.75)	10611 (6346 to 15300)	3.72 (2.23 to 5.33)	-2.22 (-2.28 to -2.16)
Low-middle SDI	1017 (623 to 1444)	1.52 (0.93 to 2.17)	2377 (1475 to 3382)	1.44 (0.89 to 2.05)	-0.24 (-0.29 to -0.18)
Low SDI	264 (158 to 382)	1.09 (0.65 to 1.58)	465 (280 to 666)	0.89 (0.53 to 1.27)	-0.72 (-0.8 to -0.64)
Middle SDI	3790 (2405 to 5216)	3.36 (2.13 to 4.63)	9983 (6154 to 14350)	3.12 (1.92 to 4.5)	-0.24 (-0.3 to -0.19)
North Africa and Middle East	379 (223 to 561)	2.08 (1.22 to 3.08)	974 (575 to 1409)	1.99 (1.17 to 2.88)	-0.07 (-0.18 to 0.03)
Oceania	4 (2 to 6)	1.38 (0.8 to 2.03)	8 (5 to 12)	1.07 (0.63 to 1.58)	-0.9 (-0.99 to -0.81)
South Asia	742 (445 to 1073)	1.2 (0.72 to 1.75)	1585 (971 to 2286)	0.93 (0.57 to 1.34)	-1.02 (-1.12 to -0.92)
Southeast Asia	946 (585 to 1341)	3.47 (2.14 to 4.93)	2924 (1783 to 4275)	3.91 (2.38 to 5.72)	0.24 (0.13 to 0.35)
Southern Latin America	319 (188 to 474)	5.37 (3.16 to 8.02)	491 (281 to 743)	4.35 (2.49 to 6.57)	-0.47 (-0.67 to -0.27)
Southern Sub-Saharan Africa	67 (40 to 98)	2.25 (1.36 to 3.33)	120 (71 to 176)	1.81 (1.07 to 2.68)	-0.76 (-0.98 to -0.53)
Tropical Latin America	355 (212 to 517)	3.47 (2.06 to 5.09)	872 (514 to 1283)	2.73 (1.61 to 4.03)	-0.88 (-0.96 to -0.79)
Western Europe	5976 (3699 to 8373)	7.7 (4.77 to 10.78)	5041 (2945 to 7330)	3.97 (2.35 to 5.74)	-2.2 (-2.25 to -2.15)
Western Sub-Saharan Africa	44 (25 to 65)	0.46 (0.27 to 0.68)	86 (49 to 125)	0.43 (0.24 to 0.62)	-0.06 (-0.14 to 0.01)

ASR: age-standardized rate; UI: uncertainty intervals; CI: confidence interval; SDI: socio-demographic index; EAPC: estimated annual percentage change.

Supplementary Table S5 Global and regional numbers and age-standardized DALY rates for liver cancer attributable to smoking in adults aged ≥60 years, with EAPC from 1990 to 2021, based on GBD 2021.

location	1990		2021		EAPC（95%CI）
	Number（95%UI）	ASR(per 100,000，95%UI)	Number（95%UI）	ASR(per 100,000，95%UI)	
Andean Latin America	498 (152 to 914)	20.84 (6.36 to 38.3)	1400 (416 to 2681)	19.43 (5.78 to 37.23)	-0.26 (-0.57 to 0.04)
Australasia	772 (247 to 1350)	24.58 (7.86 to 43)	2785 (847 to 5191)	40.43 (12.33 to 75.06)	1.32 (1.17 to 1.48)
Caribbean	754 (242 to 1337)	23.19 (7.45 to 41.19)	1293 (406 to 2297)	19.32 (6.06 to 34.3)	-1.02 (-1.3 to -0.74)
Central Asia	4771 (1561 to 8085)	79.25 (25.76 to 134.66)	7601 (2527 to 12904)	72.04 (23.8 to 122.71)	-0.42 (-0.54 to -0.31)
Central Europe	8565 (2737 to 14528)	42.27 (13.52 to 71.77)	12743 (4265 to 22003)	43.09 (14.45 to 74.26)	0.07 (-0.07 to 0.21)
Central Latin America	3235 (1065 to 5547)	33.28 (10.94 to 57.16)	7420 (2433 to 13009)	23.98 (7.85 to 42.09)	-1.18 (-1.4 to -0.96)
Central Sub-Saharan Africa	1351 (302 to 3634)	49.48 (11 to 135.74)	1797 (384 to 4588)	28.73 (6.08 to 74.24)	-2.06 (-2.32 to -1.8)
East Asia	150881 (51338 to 252510)	135.15 (45.63 to 226.94)	287079 (95507 to 498857)	100.93 (33.53 to 175.59)	-0.75 (-0.91 to -0.58)
Eastern Europe	8035 (2846 to 13352)	20.75 (7.31 to 34.57)	14325 (4946 to 23868)	29.12 (10.05 to 48.54)	1.1 (0.85 to 1.35)
Eastern Sub-Saharan Africa	3752 (1128 to 7129)	42.97 (12.87 to 81.76)	6277 (1825 to 12863)	33.12 (9.58 to 67.8)	-1.34 (-1.61 to -1.06)
Global	386715 (133546 to 630318)	76.37 (26.31 to 124.8)	708190 (240659 to 1196883)	64.33 (21.83 to 108.81)	-0.69 (-0.82 to -0.56)
High-income Asia Pacific	67341 (23523 to 109765)	257.47 (89.73 to 420.31)	60798 (18890 to 107814)	105.32 (32.78 to 185.5)	-3.85 (-4.3 to -3.4)

High-income North America	13250 (4335 to 22836)	28.81 (9.46 to 49.57)	49862 (15377 to 88781)	56.91 (17.56 to 101.22)	2.22 (2.11 to 2.33)
High-middle SDI	103319 (35274 to 171489)	78.49 (26.73 to 130.55)	180046 (59584 to 306616)	69.43 (22.95 to 118.32)	-0.38 (-0.53 to -0.24)
High SDI	115692 (40423 to 190729)	80.88 (28.3 to 133.24)	181027 (57122 to 317828)	67.17 (21.23 to 117.53)	-1.18 (-1.47 to -0.89)
Low-middle SDI	32688 (11248 to 55339)	44.24 (15.18 to 75.24)	78203 (25746 to 134645)	43.9 (14.43 to 75.6)	-0.07 (-0.12 to -0.02)
Low SDI	11261 (3540 to 20290)	41.9 (13.13 to 75.64)	17855 (5381 to 32695)	30.58 (9.19 to 56.02)	-1.27 (-1.39 to -1.16)
Middle SDI	123482 (42460 to 205971)	96.46 (33.01 to 161.16)	250608 (85314 to 431389)	73.74 (25.1 to 126.97)	-0.74 (-0.88 to -0.6)
North Africa and Middle East	10742 (3325 to 19765)	52.52 (16.21 to 96.98)	34434 (10322 to 61282)	63.23 (18.99 to 112.57)	0.75 (0.65 to 0.84)
Oceania	187 (51 to 436)	51.91 (14.11 to 120.79)	306 (89 to 655)	34.95 (10.16 to 74.65)	-1.55 (-1.74 to -1.37)
South Asia	20152 (6985 to 34031)	29.23 (10.13 to 49.52)	51381 (16356 to 88631)	27.86 (8.86 to 48.11)	-0.17 (-0.27 to -0.07)
Southeast Asia	36185 (12494 to 63134)	117.85 (40.52 to 205.65)	76119 (24412 to 137059)	91.25 (29.24 to 164.75)	-1.12 (-1.22 to -1.01)
Southern Latin America	693 (210 to 1228)	11.43 (3.46 to 20.28)	2516 (773 to 4464)	22.56 (6.94 to 39.99)	2.78 (2.52 to 3.04)
Southern Sub-Saharan Africa	1797 (475 to 3729)	55.32 (14.63 to 114.76)	3896 (1204 to 7067)	54.65 (16.87 to 99.5)	-1.05 (-1.65 to -0.44)
Tropical Latin America	3085 (1027 to 5220)	27.89 (9.26 to 47.4)	7774 (2421 to 13731)	23.84 (7.42 to 42.2)	0.01 (-0.22 to 0.23)
Western Europe	44886 (14936 to 75113)	59.69 (19.91 to 99.74)	70827 (21971 to 124798)	61.9 (19.27 to 108.28)	0.06 (-0.06 to 0.18)
Western Sub-Saharan Africa	5782 (1769 to 11029)	54.78 (16.69 to 104.63)	7560 (2161 to 13871)	34.38 (9.81 to 63.24)	-1.72 (-1.86 to -1.58)

DALYs: disability-adjusted life years; ASR: age-standardized rate; UI: uncertainty intervals; CI: confidence interval; SDI: socio-demographic index; EAPC: estimated annual percentage change.

Supplementary Table S6 Global and regional numbers and age-standardized death rates for liver cancer attributable to smoking in adults aged ≥60 years, with EAPC from 1990 to 2021, based on GBD 2021.

location	1990		2021		EAPC (95%CI)
	Number（95%UI）	ASR(per 100,000, 95%UI)	Number（95%UI）	ASR(per 100,000, 95%UI)	
Andean Latin America	24 (7 to 44)	1.02 (0.31 to 1.9)	70 (21 to 135)	0.98 (0.29 to 1.89)	-0.18 (-0.5 to 0.14)
Australasia	35 (11 to 63)	1.13 (0.36 to 1.99)	139 (42 to 263)	1.96 (0.59 to 3.68)	1.51 (1.34 to 1.68)
Caribbean	36 (12 to 65)	1.13 (0.36 to 2.03)	63 (20 to 113)	0.94 (0.29 to 1.68)	-1.06 (-1.34 to -0.77)
Central Asia	197 (64 to 335)	3.39 (1.1 to 5.79)	321 (106 to 547)	3.24 (1.06 to 5.55)	-0.21 (-0.33 to -0.09)
Central Europe	376 (120 to 641)	1.91 (0.61 to 3.26)	584 (193 to 1016)	1.95 (0.64 to 3.38)	0.07 (-0.06 to 0.21)
Central Latin America	153 (50 to 264)	1.64 (0.53 to 2.82)	364 (118 to 642)	1.2 (0.39 to 2.12)	-1.12 (-1.32 to -0.93)
Central Sub-Saharan Africa	57 (13 to 155)	2.27 (0.5 to 6.37)	76 (16 to 196)	1.32 (0.28 to 3.47)	-2.06 (-2.34 to -1.79)
East Asia	6455 (2178 to 10845)	6.17 (2.05 to 10.41)	13378 (4453 to 23292)	4.83 (1.6 to 8.41)	-0.56 (-0.73 to -0.4)
Eastern Europe	338 (118 to 564)	0.9 (0.31 to 1.5)	619 (211 to 1040)	1.28 (0.44 to 2.15)	1.14 (0.9 to 1.38)
Eastern Sub-Saharan Africa	167 (50 to 318)	2.08 (0.62 to 3.96)	283 (82 to 580)	1.6 (0.46 to 3.28)	-1.34 (-1.61 to -1.07)
Global	16951 (5814 to 27797)	3.45 (1.18 to 5.69)	33539 (11283 to 57129)	3.09 (1.04 to 5.27)	-0.49 (-0.61 to -0.36)
High-income Asia Pacific	2925 (1011 to 4802)	11.38 (3.92 to 18.72)	3424 (1058 to 6140)	5.38 (1.67 to 9.55)	-3.31 (-3.75 to -2.86)

High-income North America	630 (203 to 1096)	1.35 (0.43 to 2.34)	2357 (720 to 4246)	2.66 (0.81 to 4.79)	2.18 (2.08 to 2.29)
High-middle SDI	4482 (1518 to 7477)	3.52 (1.19 to 5.89)	8365 (2753 to 14295)	3.25 (1.07 to 5.57)	-0.25 (-0.38 to -0.11)
High SDI	5205 (1796 to 8641)	3.61 (1.25 to 5.99)	9320 (2920 to 16560)	3.32 (1.04 to 5.87)	-0.79 (-1.08 to -0.49)
Low-middle SDI	1417 (486 to 2415)	2.03 (0.69 to 3.48)	3490 (1145 to 6008)	2.04 (0.67 to 3.52)	-0.02 (-0.06 to 0.03)
Low SDI	496 (156 to 895)	1.99 (0.62 to 3.6)	809 (243 to 1481)	1.48 (0.44 to 2.71)	-1.21 (-1.32 to -1.09)
Middle SDI	5340 (1824 to 8926)	4.42 (1.5 to 7.4)	11534 (3928 to 19898)	3.51 (1.19 to 6.07)	-0.61 (-0.74 to -0.47)
North Africa and Middle East	471 (145 to 869)	2.45 (0.75 to 4.55)	1529 (460 to 2721)	2.96 (0.89 to 5.27)	0.71 (0.61 to 0.81)
Oceania	8 (2 to 18)	2.34 (0.63 to 5.43)	13 (4 to 27)	1.57 (0.45 to 3.34)	-1.56 (-1.74 to -1.38)
South Asia	867 (300 to 1470)	1.33 (0.46 to 2.27)	2327 (741 to 4020)	1.32 (0.42 to 2.28)	-0.02 (-0.13 to 0.08)
Southeast Asia	1573 (540 to 2745)	5.44 (1.86 to 9.49)	3342 (1071 to 6046)	4.27 (1.37 to 7.76)	-1.07 (-1.18 to -0.95)
Southern Latin America	31 (9 to 55)	0.51 (0.15 to 0.92)	117 (36 to 210)	1.04 (0.32 to 1.86)	2.89 (2.63 to 3.15)
Southern Sub-Saharan Africa	83 (22 to 172)	2.68 (0.71 to 5.56)	174 (53 to 317)	2.59 (0.8 to 4.76)	-1.14 (-1.75 to -0.52)
Tropical Latin America	141 (47 to 241)	1.34 (0.44 to 2.31)	363 (113 to 651)	1.14 (0.35 to 2.04)	-0.05 (-0.28 to 0.18)
Western Europe	2124 (698 to 3587)	2.77 (0.91 to 4.67)	3651 (1124 to 6529)	3 (0.93 to 5.31)	0.18 (0.06 to 0.3)
Western Sub-Saharan Africa	259 (79 to 496)	2.6 (0.79 to 4.98)	345 (98 to 635)	1.68 (0.48 to 3.11)	-1.63 (-1.77 to -1.49)

ASR: age-standardized rate; UI: uncertainty intervals; CI: confidence interval; SDI: socio-demographic index; EAPC: estimated annual percentage change.

Supplementary Table S7 Global and regional numbers and age-standardized DALY rates for pancreatic cancer attributable to smoking in adults aged ≥60 years, with EAPC from 1990 to 2021, based on GBD 2021.

location	1990		2021		EAPC （95%CI）
	Number（95%UI）	ASR(per 100,000, 95%UI)	Number（95%UI）	ASR(per 100,000, 95%UI)	
Andean Latin America	1078 (809 to 1417)	45.03 (33.75 to 59.24)	3384 (2383 to 4654)	46.88 (33 to 64.51)	0.17 (0.04 to 0.3)
Australasia	4312 (3661 to 5041)	137.14 (116.28 to 160.54)	5735 (4294 to 7494)	81.85 (61.59 to 106.54)	-1.62 (-1.7 to -1.53)
Caribbean	2738 (2212 to 3344)	84.62 (68.24 to 103.53)	4907 (3812 to 6220)	73.24 (56.9 to 92.81)	0.05 (-0.16 to 0.26)
Central Asia	2726 (2262 to 3313)	45.03 (37.27 to 54.86)	7187 (6014 to 8420)	69.43 (58.12 to 81.51)	1.75 (1.49 to 2.02)
Central Europe	34451 (30722 to 38579)	170.36 (151.63 to 191.18)	53045 (45403 to 60971)	178.87 (153.25 to 205.43)	0.28 (0.2 to 0.35)
Central Latin America	7323 (6198 to 8534)	75.77 (63.89 to 88.57)	15248 (12499 to 18424)	48.99 (40.1 to 59.27)	-1.79 (-2.01 to -1.58)
Central Sub-Saharan Africa	544 (382 to 756)	20.03 (14.03 to 28.1)	1174 (774 to 1722)	18.75 (12.31 to 27.73)	-0.17 (-0.58 to 0.24)
East Asia	113455 (91096 to 139185)	105.57 (84.74 to 129.47)	350487 (260196 to 462065)	123.5 (91.72 to 162.57)	0.65 (0.54 to 0.76)
Eastern Europe	44657 (39260 to 51040)	115.19 (101.02 to 131.75)	65946 (55616 to 77140)	133.63 (112.65 to 156.36)	0.41 (0.28 to 0.53)
Eastern Sub-Saharan Africa	1732 (1304 to 2215)	19.99 (15.04 to 25.53)	3470 (2623 to 4645)	18.43 (13.92 to 24.58)	-0.54 (-0.66 to -0.43)
Global	567153 (510579 to 625194)	114.91 (103.09 to 127.03)	1102207 (943669 to 1273484)	100.44 (85.88 to 116.2)	-0.37 (-0.42 to -0.32)
High-income Asia Pacific	53280 (47386 to 59306)	207.74 (184.39 to 231.69)	84004 (66727 to 102972)	143.8 (115.88 to 174.58)	-1.17 (-1.25 to -1.1)

High-income North America	91905 (80327 to 105212)	198.65 (173.82 to 227.03)	142948 (115750 to 174051)	161.86 (131.31 to 196.7)	-0.65 (-0.7 to -0.61)
High-middle SDI	176992 (157768 to 196424)	136.23 (121.16 to 151.43)	366946 (305273 to 439290)	141.67 (117.82 to 169.57)	0.21 (0.15 to 0.27)
High SDI	278064 (248398 to 308840)	192.61 (172.09 to 213.87)	425650 (360004 to 496728)	157.2 (133.68 to 182.54)	-0.56 (-0.61 to -0.52)
Low-middle SDI	20591 (16173 to 25536)	28.56 (22.41 to 35.38)	60901 (52738 to 70716)	34.68 (29.96 to 40.29)	0.62 (0.58 to 0.66)
Low SDI	4483 (3263 to 5622)	16.78 (12.2 to 21.1)	9349 (7390 to 11809)	16.15 (12.74 to 20.4)	-0.26 (-0.39 to -0.13)
Middle SDI	86223 (73905 to 100163)	69.52 (59.6 to 80.68)	238135 (195439 to 287953)	70.67 (57.97 to 85.48)	0.09 (-0.01 to 0.18)
North Africa and Middle East	11383 (8591 to 14803)	56.65 (42.54 to 73.77)	41208 (32749 to 51536)	77.39 (61.35 to 96.77)	1.09 (0.95 to 1.24)
Oceania	116 (87 to 154)	33.16 (24.94 to 44.36)	297 (222 to 397)	34.72 (25.82 to 46.51)	0.07 (-0.02 to 0.15)
South Asia	14769 (10955 to 18944)	21.92 (16.18 to 28.16)	38588 (31035 to 47459)	21.25 (17.09 to 26.14)	-0.3 (-0.46 to -0.13)
Southeast Asia	14886 (12351 to 17819)	49.78 (41.25 to 59.61)	50091 (41209 to 61035)	61.58 (50.57 to 75.1)	0.55 (0.48 to 0.63)
Southern Latin America	10009 (7849 to 12591)	165.3 (129.35 to 208.33)	15429 (12227 to 19145)	138.61 (109.98 to 171.79)	-0.41 (-0.56 to -0.26)
Southern Sub-Saharan Africa	2244 (1717 to 3107)	69.76 (53.26 to 96.8)	4986 (3862 to 6229)	69.64 (53.69 to 87.45)	-0.15 (-0.39 to 0.1)
Tropical Latin America	13701 (11470 to 16169)	125.02 (103.98 to 148.53)	30202 (24706 to 36155)	92.85 (75.74 to 111.44)	-1.11 (-1.26 to -0.95)
Western Europe	141121 (124889 to 157281)	186.21 (165.03 to 207.26)	181688 (154305 to 212454)	157.75 (135.07 to 182.97)	-0.28 (-0.35 to -0.2)
Western Sub-Saharan Africa	723 (576 to 889)	6.92 (5.51 to 8.54)	2183 (1700 to 2741)	9.87 (7.68 to 12.42)	1.27 (1.2 to 1.34)

DALYs: disability-adjusted life years; ASR: age-standardized rate; UI: uncertainty intervals; CI: confidence interval; SDI: socio-demographic index; EAPC: estimated annual percentage change.

Supplementary Table S8 Global and regional numbers and age-standardized death rates for pancreatic cancer attributable to smoking in adults aged ⩾60 years, with EAPC from 1990 to 2021, based on GBD 2021.

location	1990		2021		EAPC（95%CI）
	Number（95%UI）	ASR(per 100,000, 95%UI)	Number（95%UI）	ASR(per 100,000, 95%UI)	
Andean Latin America	51 (38 to 68)	2.2 (1.64 to 2.9)	166 (116 to 230)	2.32 (1.63 to 3.22)	0.23 (0.09 to 0.37)
Australasia	206 (173 to 244)	6.58 (5.51 to 7.81)	303 (221 to 403)	4.18 (3.08 to 5.53)	-1.41 (-1.49 to -1.34)
Caribbean	136 (108 to 168)	4.27 (3.4 to 5.31)	243 (187 to 310)	3.61 (2.78 to 4.61)	-0.01 (-0.23 to 0.21)
Central Asia	114 (94 to 139)	1.95 (1.61 to 2.4)	311 (261 to 365)	3.22 (2.69 to 3.79)	2.08 (1.8 to 2.37)
Central Europe	1529 (1355 to 1724)	7.79 (6.88 to 8.81)	2447 (2078 to 2835)	8.13 (6.91 to 9.41)	0.24 (0.16 to 0.31)
Central Latin America	352 (295 to 414)	3.8 (3.17 to 4.49)	726 (591 to 884)	2.38 (1.93 to 2.9)	-1.91 (-2.13 to -1.69)
Central Sub-Saharan Africa	23 (16 to 32)	0.93 (0.64 to 1.31)	50 (33 to 73)	0.86 (0.56 to 1.29)	-0.2 (-0.62 to 0.22)
East Asia	5083 (4085 to 6232)	5.15 (4.13 to 6.32)	17045 (12701 to 22369)	6.19 (4.62 to 8.11)	0.72 (0.63 to 0.8)
Eastern Europe	1870 (1634 to 2147)	4.97 (4.33 to 5.71)	2839 (2384 to 3338)	5.83 (4.89 to 6.86)	0.48 (0.35 to 0.6)
Eastern Sub-Saharan Africa	78 (59 to 99)	0.98 (0.74 to 1.25)	158 (119 to 210)	0.9 (0.68 to 1.2)	-0.52 (-0.63 to -0.41)
Global	26627 (23799 to 29515)	5.65 (5.02 to 6.29)	54271 (46026 to 63341)	5.03 (4.26 to 5.88)	-0.33 (-0.36 to -0.29)
High-income Asia Pacific	2564 (2264 to 2878)	10.27 (9.04 to 11.57)	4749 (3691 to 5904)	7.4 (5.86 to 9.09)	-1.08 (-1.17 to -0.99)

High-income North America	4493 (3879 to 5205)	9.55 (8.25 to 11.05)	7201 (5719 to 8946)	8.07 (6.42 to 10)	-0.53 (-0.58 to -0.48)
High-middle SDI	7952 (7052 to 8862)	6.38 (5.64 to 7.13)	17512 (14481 to 21034)	6.83 (5.64 to 8.2)	0.3 (0.24 to 0.36)
High SDI	13629 (12051 to 15290)	9.39 (8.3 to 10.54)	22121 (18361 to 26277)	7.84 (6.56 to 9.26)	-0.51 (-0.54 to -0.47)
Low-middle SDI	921 (723 to 1140)	1.37 (1.07 to 1.7)	2803 (2417 to 3253)	1.68 (1.44 to 1.95)	0.66 (0.62 to 0.69)
Low SDI	199 (144 to 250)	0.8 (0.58 to 1.02)	429 (339 to 542)	0.8 (0.62 to 1)	-0.13 (-0.26 to -0.01)
Middle SDI	3890 (3338 to 4511)	3.38 (2.9 to 3.91)	11348 (9293 to 13756)	3.5 (2.86 to 4.24)	0.11 (0.03 to 0.19)
North Africa and Middle East	510 (383 to 664)	2.74 (2.04 to 3.57)	1904 (1506 to 2382)	3.81 (3 to 4.76)	1.17 (1.03 to 1.32)
Oceania	5 (4 to 7)	1.55 (1.16 to 2.08)	13 (9 to 17)	1.6 (1.18 to 2.15)	0.04 (-0.04 to 0.11)
South Asia	651 (480 to 837)	1.04 (0.76 to 1.34)	1804 (1451 to 2216)	1.05 (0.84 to 1.29)	-0.15 (-0.3 to -0.01)
Southeast Asia	672 (556 to 805)	2.41 (1.99 to 2.89)	2283 (1873 to 2786)	3 (2.45 to 3.67)	0.56 (0.48 to 0.64)
Southern Latin America	451 (350 to 574)	7.55 (5.83 to 9.65)	719 (561 to 907)	6.39 (4.99 to 8.05)	-0.35 (-0.51 to -0.2)
Southern Sub-Saharan Africa	105 (80 to 147)	3.48 (2.64 to 4.85)	221 (170 to 278)	3.28 (2.5 to 4.17)	-0.36 (-0.61 to -0.11)
Tropical Latin America	637 (527 to 761)	6.15 (5.04 to 7.44)	1435 (1153 to 1749)	4.5 (3.6 to 5.5)	-1.14 (-1.28 to -0.99)
Western Europe	7064 (6183 to 7959)	9.16 (8.02 to 10.31)	9555 (7961 to 11381)	7.76 (6.54 to 9.14)	-0.31 (-0.38 to -0.25)
Western Sub-Saharan Africa	33 (26 to 40)	0.34 (0.27 to 0.42)	99 (77 to 124)	0.48 (0.37 to 0.61)	1.3 (1.24 to 1.36)

ASR: age-standardized rate; UI: uncertainty intervals; CI: confidence interval; SDI: socio-demographic index; EAPC: estimated annual percentage change

Supplementary Table S9 Global and regional numbers and age-standardized DALY rates for stomach cancer attributable to smoking in adults aged ≥60 years, with EAPC from 1990 to 2021, based on GBD 2021.

location	1990		2021		EAPC（95%CI）
	Number（95%UI）	ASR(per 100,000, 95%UI)	Number（95%UI）	ASR(per 100,000, 95%UI)	
Andean Latin America	4080 (3115 to 5275)	171.72 (130.78 to 222.45)	6537 (4613 to 9102)	90.93 (64.15 to 126.63)	-2.17 (-2.3 to -2.03)
Australasia	3027 (2354 to 3800)	96.34 (74.81 to 121.17)	1792 (1307 to 2423)	24.98 (18.32 to 33.54)	-4.44 (-4.56 to -4.32)
Caribbean	3499 (2748 to 4434)	109.29 (85.58 to 138.82)	3763 (2786 to 4878)	56.1 (41.53 to 72.69)	-2.17 (-2.24 to -2.1)
Central Asia	17109 (13769 to 21137)	289.92 (232.61 to 359.1)	13986 (11134 to 17268)	139.63 (110.89 to 172.48)	-1.91 (-2.08 to -1.74)
Central Europe	59542 (49182 to 70765)	300.73 (247.9 to 358.14)	34783 (27840 to 42481)	115.7 (92.69 to 141.14)	-3.12 (-3.23 to -3)
Central Latin America	15700 (12660 to 19213)	165.29 (132.81 to 202.82)	16776 (12870 to 21070)	54.38 (41.7 to 68.36)	-3.99 (-4.14 to -3.83)
Central Sub-Saharan Africa	1302 (805 to 1904)	48.5 (29.84 to 70.81)	1845 (1168 to 2672)	29.8 (18.89 to 43.12)	-1.53 (-1.73 to -1.33)
East Asia	755356 (562702 to 989289)	707.07 (525.66 to 929.81)	1049750 (735631 to 1452312)	373.05 (261.67 to 516.19)	-1.91 (-2.04 to -1.78)
Eastern Europe	134108 (110890 to 160091)	350.76 (289.39 to 419.83)	73333 (58580 to 89792)	150.39 (120.06 to 184.21)	-3.01 (-3.21 to -2.81)
Eastern Sub-Saharan Africa	4418 (3054 to 5807)	50.91 (35.34 to 66.99)	5045 (3684 to 6479)	26.84 (19.6 to 34.49)	-2.3 (-2.39 to -2.22)
Global	1628174 (1321846 to 1973517)	330.97 (268.35 to 401.62)	1674301 (1282517 to 2170914)	153.26 (117.34 to 198.66)	-2.48 (-2.53 to -2.44)
High-income Asia Pacific	182818 (146577 to 224819)	720.7 (576.99 to 887.47)	105041 (78839 to 135902)	164.55 (124.79 to 211.15)	-4.99 (-5.07 to -4.91)
High-income North America	56163 (44747 to 68360)	120.29 (95.85 to 146.28)	37845 (28827 to 48297)	42.57 (32.47 to 54.23)	-3.5 (-3.56 to -3.43)

High-middle SDI	585436 (470438 to 708424)	456.32 (366.14 to 553.25)	616473 (459337 to 822949)	238.9 (177.87 to 318.91)	-2.05 (-2.12 to -1.98)
High SDI	425300 (353033 to 504555)	293.18 (243.33 to 347.87)	246067 (193907 to 304639)	87.27 (69.12 to 107.61)	-4.1 (-4.17 to -4.02)
Low-middle SDI	83899 (64693 to 108134)	117.37 (90.42 to 151.35)	116605 (89454 to 147736)	67.33 (51.51 to 85.34)	-1.73 (-1.8 to -1.67)
Low SDI	18544 (12910 to 24079)	69.85 (48.64 to 90.8)	24917 (16363 to 31919)	43.6 (28.49 to 55.94)	-1.54 (-1.63 to -1.45)
Middle SDI	513646 (394201 to 667574)	414.62 (318.29 to 540.31)	669365 (485696 to 903577)	201.65 (146.34 to 272.14)	-2.22 (-2.3 to -2.13)
North Africa and Middle East	39227 (26770 to 52346)	201.55 (137.47 to 269.59)	55068 (34371 to 71214)	108.37 (67.72 to 140.38)	-2.01 (-2.08 to -1.95)
Oceania	453 (296 to 634)	131.13 (86.36 to 183.49)	675 (468 to 969)	80.02 (55.58 to 115.09)	-1.73 (-1.81 to -1.65)
South Asia	58423 (43566 to 79099)	87.04 (64.72 to 118.14)	83201 (60419 to 112138)	46.18 (33.44 to 62.22)	-1.98 (-2.09 to -1.88)
Southeast Asia	41718 (30007 to 53393)	141.23 (101.83 to 180.97)	57009 (43612 to 74376)	71.37 (54.51 to 93.33)	-2.54 (-2.66 to -2.43)
Southern Latin America	11172 (8516 to 14176)	185.67 (141.12 to 236.25)	9576 (7252 to 12184)	85.66 (64.95 to 108.84)	-2.42 (-2.58 to -2.26)
Southern Sub-Saharan Africa	2342 (1637 to 3058)	73.6 (51.36 to 96.35)	2953 (2151 to 3825)	41.5 (30.06 to 54.03)	-2.03 (-2.31 to -1.75)
Tropical Latin America	36995 (28553 to 46684)	344.94 (264.93 to 437.86)	29107 (22523 to 36822)	90.48 (69.89 to 114.67)	-4.57 (-4.71 to -4.44)
Western Europe	198227 (160861 to 235412)	256.62 (208.45 to 304.62)	82349 (64372 to 101685)	67.25 (53.02 to 82.43)	-4.32 (-4.39 to -4.25)
Western Sub-Saharan Africa	2497 (1842 to 3281)	23.96 (17.71 to 31.55)	3864 (2660 to 5043)	17.51 (12.1 to 22.91)	-0.81 (-0.89 to -0.74)

DALYs: disability-adjusted life years; ASR: age-standardized rate; UI: uncertainty intervals; CI: confidence interval; SDI: socio-demographic index; EAPC: estimated annual percentage change.

Supplementary Table S10 Global and regional numbers and age-standardized death rates for stomach cancer attributable to smoking in adults aged ≥60 years, with EAPC from 1990 to 2021, based on GBD 2021.

location	1990		2021		EAPC (95%CI)
	Number（95%UI）	ASR(per 100,000, 95%UI)	Number（95%UI）	ASR(per 100,000, 95%UI)	
Andean Latin America	199 (151 to 259)	8.64 (6.51 to 11.28)	334 (235 to 467)	4.71 (3.31 to 6.58)	-2.07 (-2.22 to -1.93)
Australasia	155 (119 to 197)	4.99 (3.83 to 6.36)	104 (75 to 145)	1.4 (1.01 to 1.92)	-4.16 (-4.28 to -4.03)
Caribbean	181 (140 to 232)	5.82 (4.49 to 7.5)	195 (143 to 256)	2.9 (2.12 to 3.79)	-2.25 (-2.33 to -2.16)
Central Asia	740 (593 to 917)	13.05 (10.42 to 16.22)	625 (496 to 772)	6.7 (5.3 to 8.27)	-1.59 (-1.79 to -1.39)
Central Europe	2802 (2298 to 3352)	14.63 (11.97 to 17.56)	1725 (1371 to 2123)	5.66 (4.5 to 6.96)	-3.1 (-3.22 to -2.98)
Central Latin America	786 (628 to 968)	8.74 (6.95 to 10.8)	842 (643 to 1064)	2.79 (2.13 to 3.53)	-4.07 (-4.22 to -3.92)
Central Sub-Saharan Africa	56 (34 to 81)	2.27 (1.39 to 3.31)	79 (50 to 114)	1.39 (0.88 to 2.01)	-1.56 (-1.77 to -1.35)
East Asia	34237 (25506 to 45027)	34.8 (25.81 to 46.05)	53000 (37204 to 73069)	19.56 (13.75 to 26.95)	-1.71 (-1.85 to -1.57)
Eastern Europe	5825 (4786 to 6999)	15.7 (12.87 to 18.92)	3331 (2646 to 4110)	6.94 (5.51 to 8.56)	-2.85 (-3.05 to -2.65)
Eastern Sub-Saharan Africa	197 (137 to 259)	2.48 (1.73 to 3.27)	229 (168 to 295)	1.32 (0.96 to 1.7)	-2.28 (-2.36 to -2.19)
Global	76831 (62210 to 93424)	16.34 (13.2 to 19.89)	85604 (65326 to 110850)	7.99 (6.09 to 10.34)	-2.32 (-2.37 to -2.27)
High-income Asia Pacific	9137 (7286 to 11276)	37.28 (29.65 to 46.09)	6629 (4896 to 8685)	9.36 (7.01 to 12.14)	-4.71 (-4.8 to -4.63)
High-income North America	2875 (2270 to 3536)	6.07 (4.79 to 7.46)	2042 (1526 to 2650)	2.27 (1.7 to 2.94)	-3.32 (-3.39 to -3.25)
High-middle SDI	27038 (21674 to 32837)	22.01 (17.6 to 26.81)	31047 (22956 to 41513)	12.19 (9 to 16.3)	-1.87 (-1.95 to -1.79)

High SDI	21821 (17950 to 26016)	14.99 (12.32 to 17.88)	14405 (11167 to 18070)	4.86 (3.8 to 6.07)	-3.82 (-3.89 to -3.75)
Low-middle SDI	3780 (2912 to 4877)	5.72 (4.4 to 7.38)	5502 (4198 to 6976)	3.37 (2.56 to 4.27)	-1.63 (-1.7 to -1.56)
Low SDI	823 (573 to 1069)	3.38 (2.36 to 4.4)	1162 (757 to 1490)	2.2 (1.42 to 2.82)	-1.35 (-1.44 to -1.26)
Middle SDI	23304 (17908 to 30416)	20.11 (15.45 to 26.32)	33444 (24267 to 45043)	10.52 (7.63 to 14.16)	-2.02 (-2.11 to -1.93)
North Africa and Middle East	1829 (1247 to 2448)	10.21 (6.96 to 13.71)	2745 (1716 to 3564)	5.82 (3.64 to 7.57)	-1.79 (-1.85 to -1.72)
Oceania	19 (13 to 27)	6.22 (4.13 to 8.7)	29 (20 to 42)	3.77 (2.62 to 5.43)	-1.75 (-1.84 to -1.67)
South Asia	2572 (1912 to 3492)	4.17 (3.08 to 5.67)	3924 (2834 to 5281)	2.31 (1.66 to 3.11)	-1.81 (-1.91 to -1.7)
Southeast Asia	1911 (1379 to 2450)	6.96 (5.04 to 8.94)	2666 (2034 to 3487)	3.6 (2.73 to 4.71)	-2.48 (-2.59 to -2.36)
Southern Latin America	519 (391 to 666)	8.81 (6.59 to 11.35)	465 (347 to 601)	4.11 (3.07 to 5.3)	-2.35 (-2.51 to -2.19)
Southern Sub-Saharan Africa	112 (78 to 147)	3.76 (2.61 to 4.96)	132 (95 to 172)	1.97 (1.41 to 2.6)	-2.3 (-2.58 to -2.02)
Tropical Latin America	1790 (1369 to 2282)	17.82 (13.54 to 22.92)	1473 (1126 to 1886)	4.68 (3.57 to 6.01)	-4.53 (-4.66 to -4.4)
Western Europe	10774 (8662 to 12891)	13.73 (11.05 to 16.44)	4859 (3729 to 6093)	3.66 (2.84 to 4.55)	-4.28 (-4.35 to -4.21)
Western Sub-Saharan Africa	113 (84 to 149)	1.16 (0.86 to 1.54)	175 (121 to 229)	0.85 (0.59 to 1.12)	-0.8 (-0.87 to -0.73)

ASR: age-standardized rate; UI: uncertainty intervals; CI: confidence interval; SDI: socio-demographic index; EAPC: estimated annual percentage change.

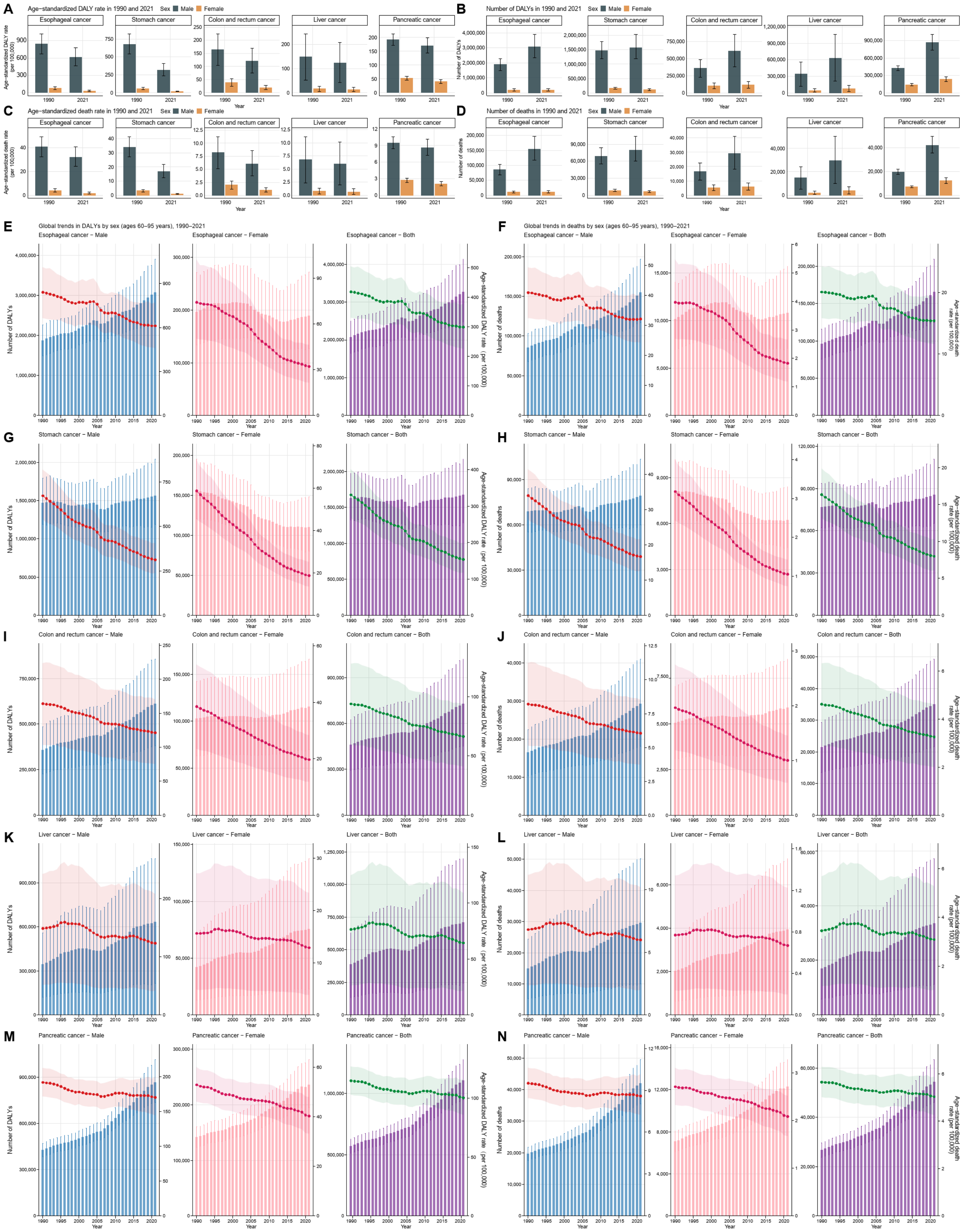

Supplementary Figure S1. Global trends in smoking-attributable deaths and DALYs from five digestive cancers in adults aged ⩾60 years, by sex, 1990－2021. (A) Age-standardized DALY rate. (B) Number of DALYs. (C) Age-standardized death rate. (D) Number of deaths. Temporal trends from 1990 to 2021 in (E, G, I, K, M) the number of DALYs and the age-standardized DALY rate, and in (F, H, J, L, N) the number of deaths and the age-standardized death rate for esophageal, stomach, colon and rectum, liver, and pancreatic cancers, respectively, among adults aged 60 years and older, shown separately for males, females, and both sexes combined. All rates are expressed per 100,000

population. DALYs: disability-adjusted life years.

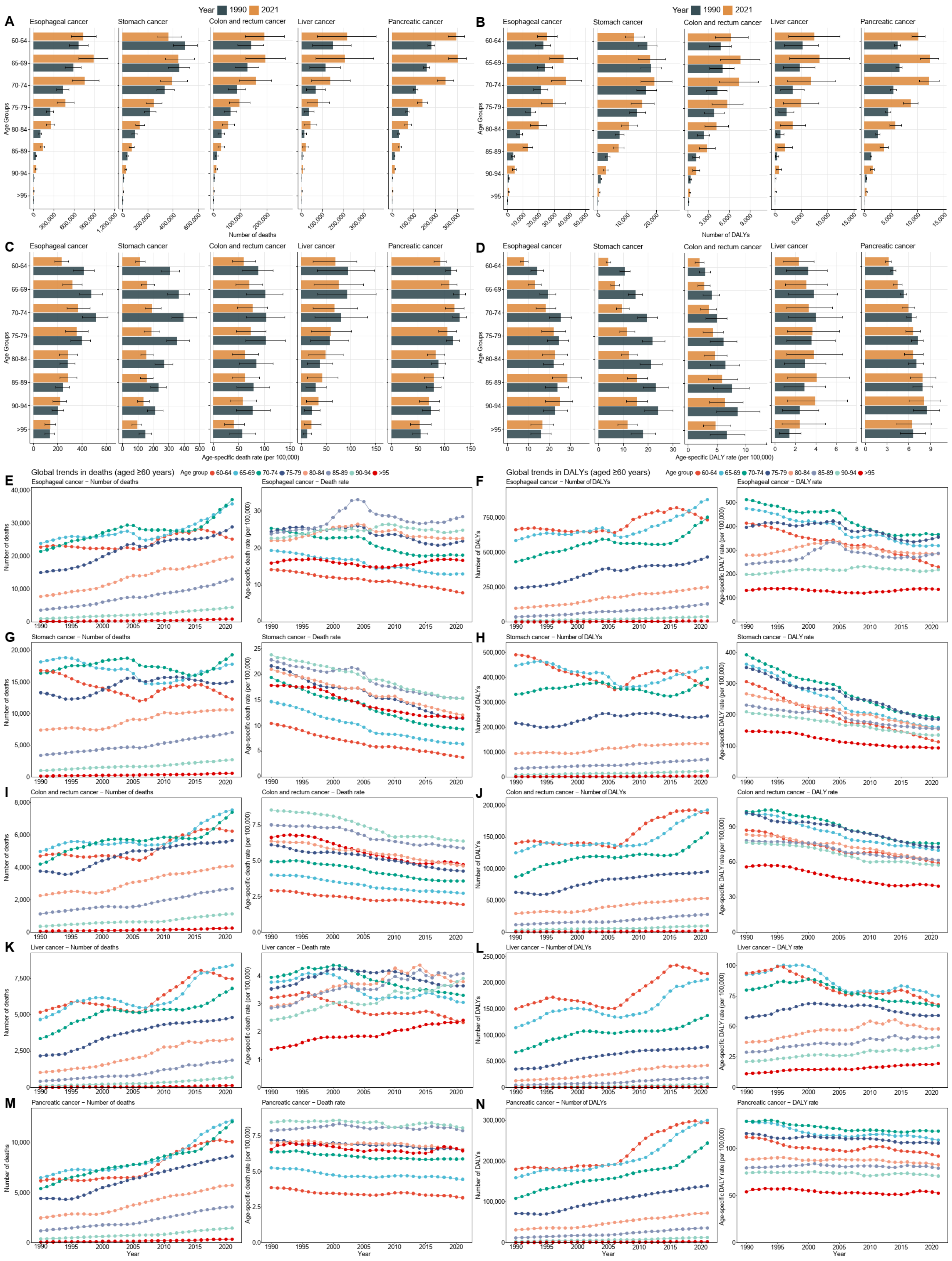

Supplementary Figure S2. Global age-specific patterns and temporal trends in smoking-attributable deaths and DALYs from five digestive cancers in adults aged ⩾60 years, 1990–2021. (A) Age-specific number of deaths in 1990 and 2021. (B) Age-specific number of DALYs in 1990 and 2021. (C) Age-

specific death rates. (D) Age-specific DALY rates. Temporal trends from 1990 to 2021 in (E, G, I, K, M) the age-specific number of deaths and the age-specific death rate, and in (F, H, J, L, N) the age-specific number of DALYs and the age-specific DALY rate for esophageal, stomach, colon and rectum, liver, and pancreatic cancers, respectively, among adults aged 60 years and older, shown for both sexes combined and stratified by 5-year age groups (60 – 64, 65 – 69, 70 – 74, 75 – 79, 80 – 84, 85 – 89, 90 – 94, and ⩾95 years). All rates are expressed per 100,000 population. Error bars indicate 95% UIs. DALYs: disability-adjusted life years; UI: uncertainty interval.

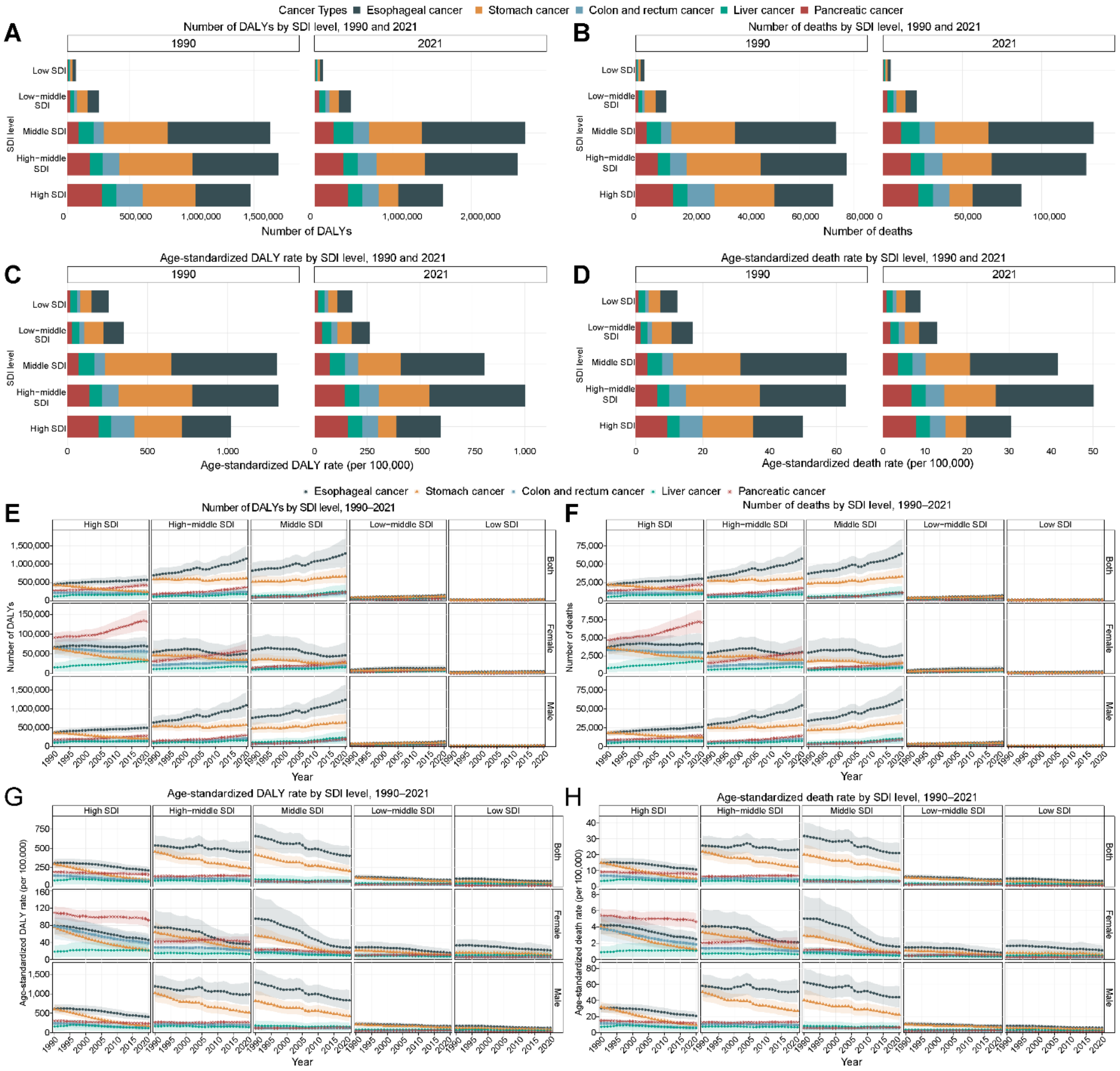

Supplementary Figure S3. SDI-specific patterns and temporal trends in smoking-attributable deaths and DALYs from five digestive cancers in adults aged ⩾60 years, by SDI level, 1990 – 2021. For 1990 and 2021, (A – D) show, by SDI level and cancer type: (A) the number of DALYs, (B) the number of deaths, (C) the age-standardized DALY rate, and (D) the age-standardized death rate. (E – H) display temporal trends from 1990 to 2021 in (E) the number of DALYs, (F) the number of deaths, (G) the age-standardized DALY rate, and (H) the age-standardized death rate across SDI levels (high, high-middle, middle, low-middle, and low SDI) for esophageal, stomach, colon and rectum, liver, and pancreatic cancers in adults aged 60 years and older. All rates are expressed per 100,000 population.

DALYs: disability-adjusted life years; SDI: Socio-demographic Index.

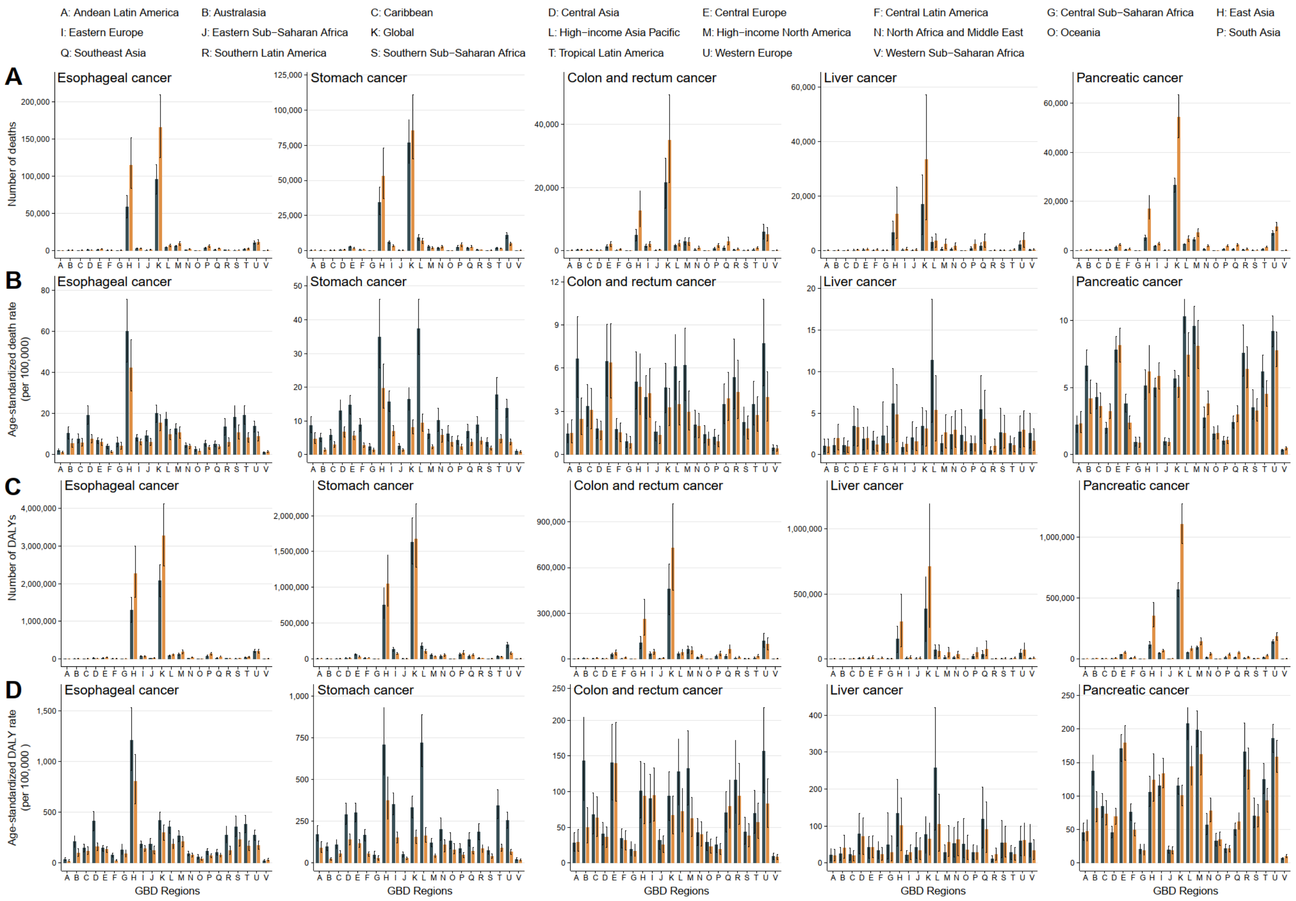

Supplementary Figure S4. Global ecological comparison of smoking-attributable deaths and DALYs from five digestive cancers in adults aged ≥60 years across 204 countries and territories, stratified by GBD region, in 1990 and 2021. Panels (A − D) show, by GBD region and cancer type, (A) the number of deaths, (B) the age-standardized death rate, (C) the number of DALYs, and (D) the age-standardized DALY rate. All rates are expressed per 100,000 population. DALYs: disability-adjusted life years; GBD: Global Burden of Disease.

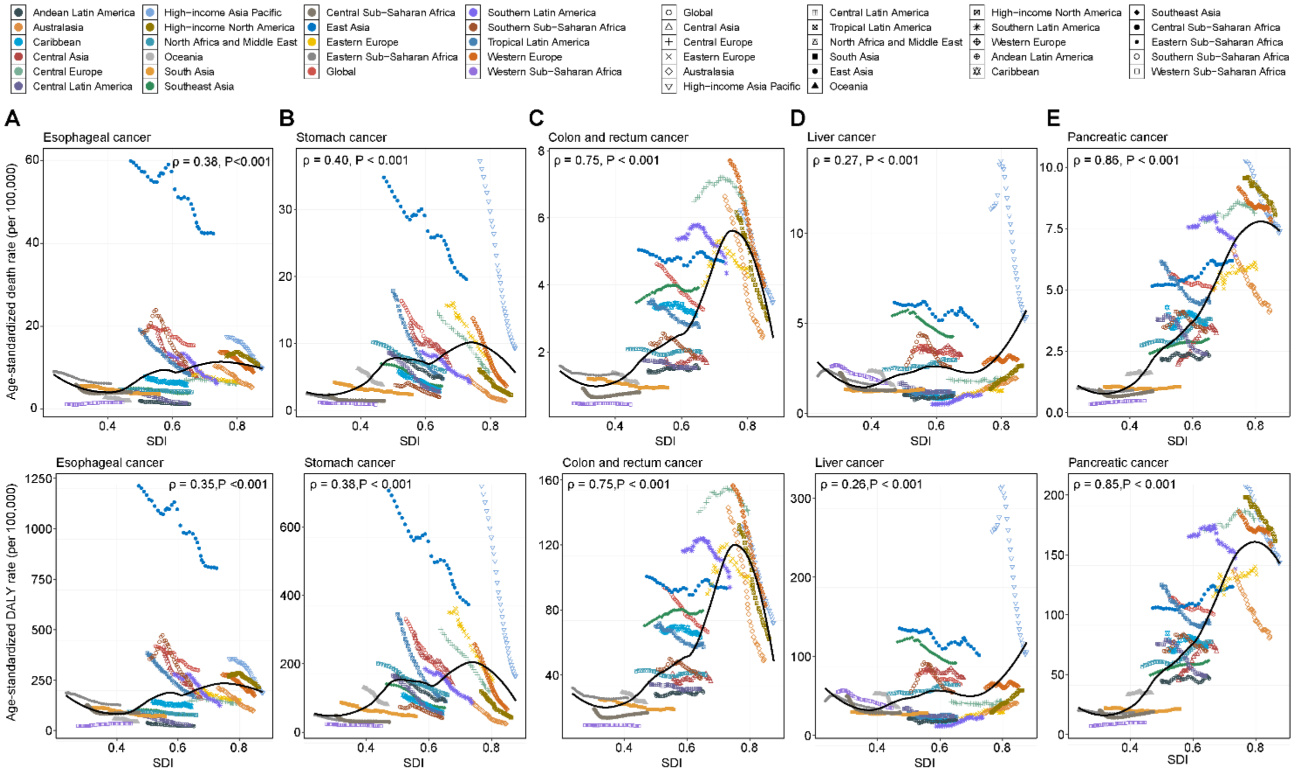

Supplementary Figure S5. Global associations between the SDI and age-standardized rates of smoking-attributable deaths and DALYs from five digestive cancers in adults aged ⩾60 years across 204 countries and territories, 1990 – 2021. (A) Esophageal cancer; (B) stomach cancer; (C) colon and rectum cancer; (D) liver cancer; (E) pancreatic cancer. For each cancer, the upper row shows the association between SDI and the age-standardized death rate, and the lower row shows the association between SDI and the age-standardized DALY rate. Each colored trajectory represents a GBD region over time (1990 – 2021), and the solid black curve indicates the overall smoothed association (locally weighted scatterplot smoothing). Spearman rank correlation coefficients (ρ) and corresponding *P* values (where shown) summarize the monotonic SDI – burden associations. All rates are expressed per 100,000 population. DALYs: disability-adjusted life years; SDI: Socio-demographic Index; GBD: Global Burden of Disease.

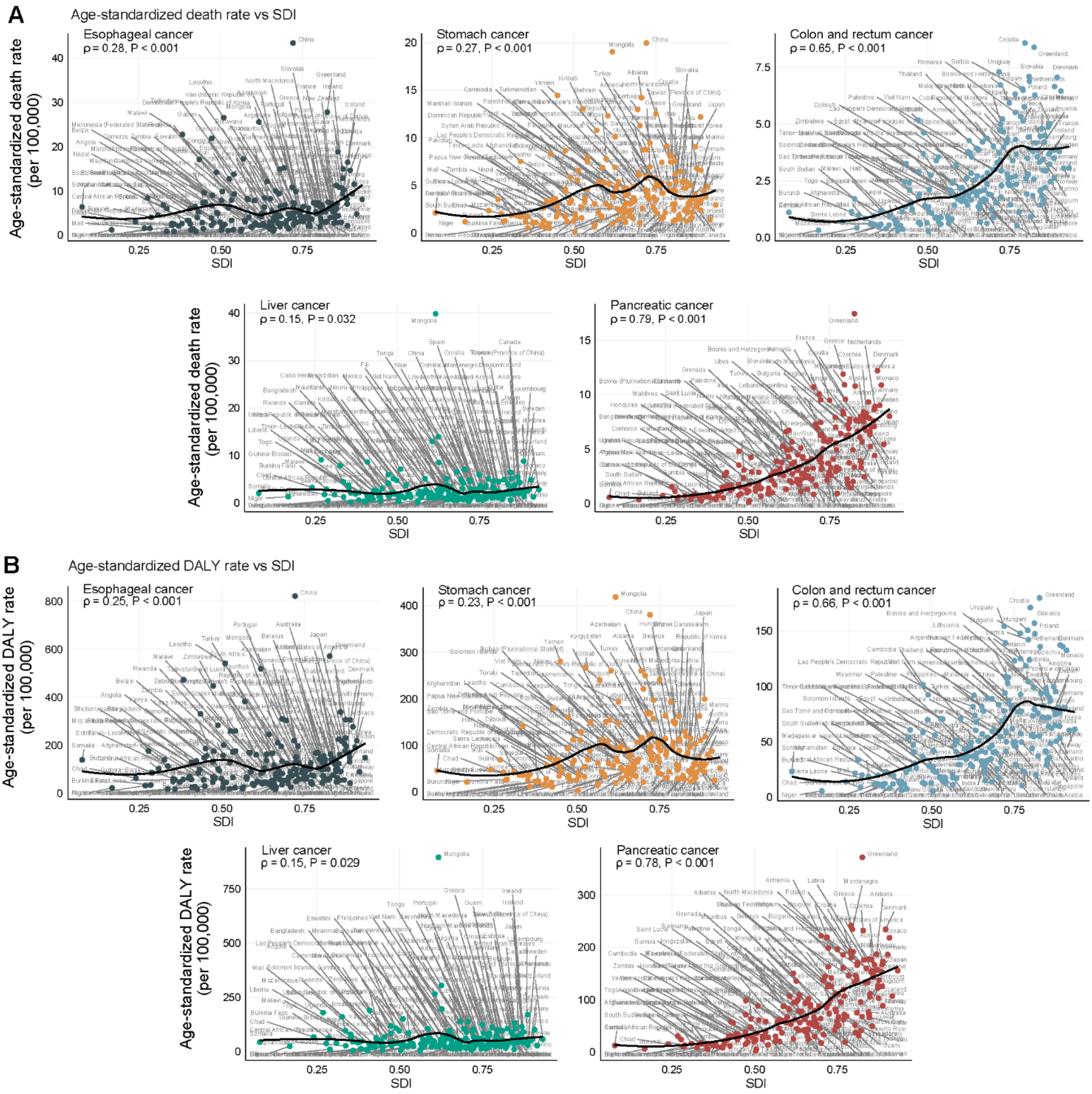

Supplementary Figure S6. Associations between the SDI and age-standardized rates of smoking-attributable deaths and DALYs from five digestive cancers in adults aged ⩾60 years across 204 countries and territories, 1990 – 2021. (A) Associations between SDI and the age-standardized death rates for esophageal, stomach, colon and rectum, liver, and pancreatic cancers. (B) Associations between SDI and age-standardized DALY rates for the same cancers. Each point represents a country or territory, colored by GBD region. Where fitted lines are shown, they represent locally weighted smoothed relationships between SDI and the corresponding age-standardized rate. Spearman rank correlation coefficients (ρ) and *P* values (where displayed) quantify the strength and direction of SDI – burden associations. All rates are expressed per 100,000 population. DALYs: disability-adjusted life years; SDI: Socio-demographic Index; GBD: Global Burden of Disease.

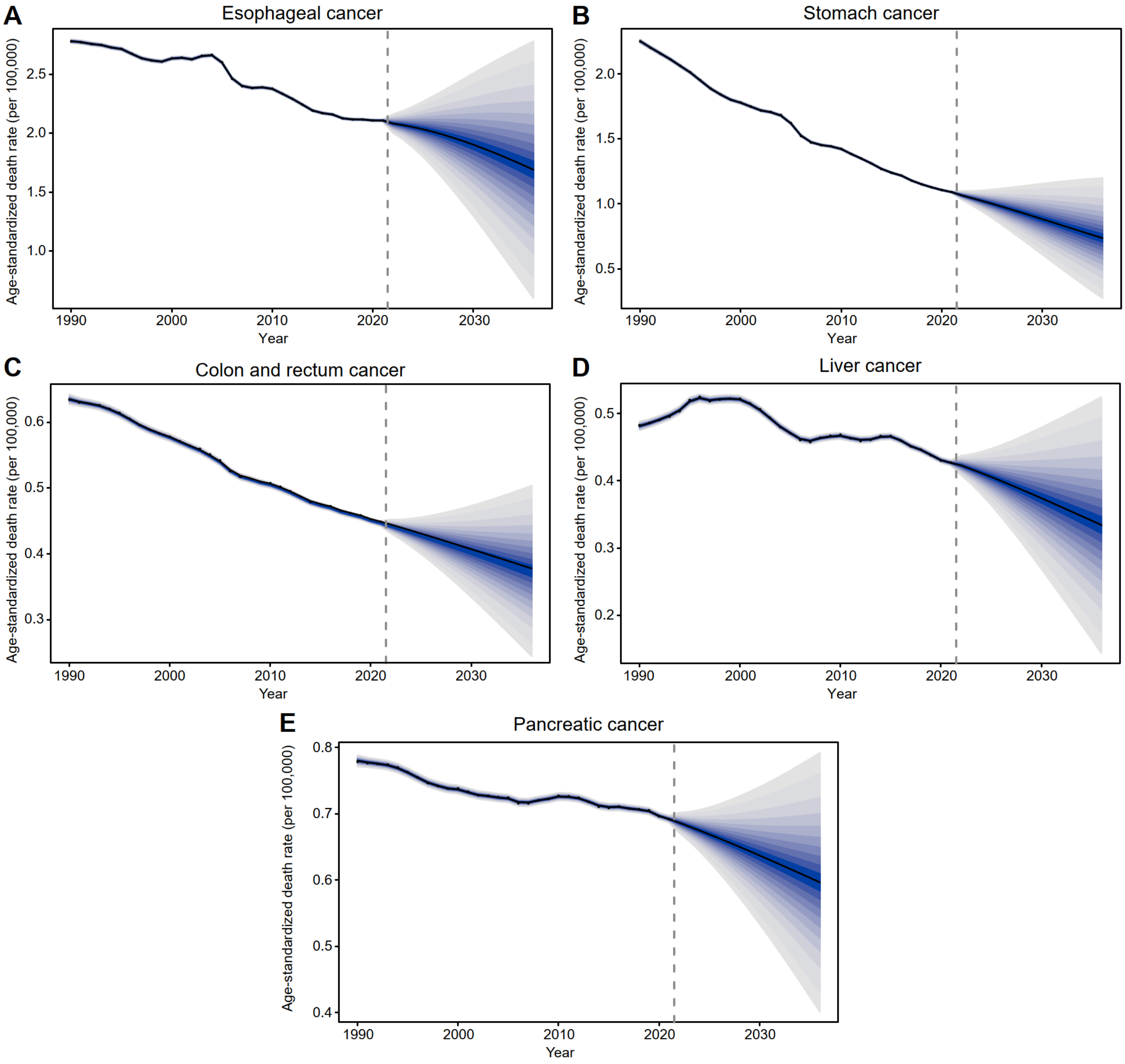

Supplementary Figure S7. Global trends and projections of smoking-attributable age-standardized death rates for five digestive cancers in adults aged ≥60 years across 204 countries and territories, 1990－2036. (A) Esophageal cancer; (B) stomach cancer; (C) colon and rectum cancer; (D) liver cancer; (E) pancreatic cancer. Dark blue solid lines show observed age-standardized death rates from GBD 2021 for 1990－2021. For 2022－2036, dark blue lines represent BAPC median projections, and shaded bands indicate 95% UIs around the projected rates. The vertical dashed line indicates 2021. All rates are expressed per 100,000 population. BAPC: Bayesian age－period－cohort; UI: uncertainty interval.

Supplementary Equation S1.

EAPC = 100 × (exp(β) − 1), where β is the slope from linear regression of ln(age-standardized rate) on calendar year. The 95% confidence intervals (CIs) for EAPCs were derived from the CIs of β [23].

Supplementary Equation S2.

Change decomposition in the number of cases. The change in the number of cases between baseline (0) and follow-up (1) was expressed as:

$$\Delta N = N_1 - N_0 = \Delta N_{pop} + \Delta N_{age} + \Delta N_{epi}$$

where $N0$ and N_1 denote the numbers of cases at baseline and follow-up, respectively, and ΔN_{pop}, ΔN_{age}, and ΔN_{epi} denote the contributions of population growth, aging, and epidemiological change, respectively.

© **2026 Wang X. and Huang L.**
